# Twin boundary defect engineering improves lithium-ion diffusion for fast-charging spinel cathode materials

**DOI:** 10.1038/s41467-021-23375-7

**Published:** 2021-05-25

**Authors:** Rui Wang, Xin Chen, Zhongyuan Huang, Jinlong Yang, Fusheng Liu, Mihai Chu, Tongchao Liu, Chaoqi Wang, Weiming Zhu, Shuankui Li, Shunning Li, Jiaxin Zheng, Jie Chen, Lunhua He, Lei Jin, Feng Pan, Yinguo Xiao

**Affiliations:** 1 School of Advanced Materials, Peking University, Shenzhen Graduate School, Shenzhen, China; 2 College of Materials Science and Engineering, Shenzhen University, Shenzhen, China; 3 Institute of High Energy Physics, Chinese Academy of Sciences, Beijing, China; 4 Spallation Neutron Source Science Center, Dongguan, China; 5 Beijing National Laboratory for Condensed Matter Physics, Institute of Physics, Chinese Academy of Sciences, Beijing, China; 6 Songshan Lake Materials Laboratory, Dongguan, China; 7 Ernst Ruska-Centre for Microscopy and Spectroscopy with Electrons, Forschungszentrum Jülich GmbH, Jülich, Germany

**Keywords:** Energy, Batteries, Batteries

## Abstract

Defect engineering on electrode materials is considered an effective approach to improve the electrochemical performance of batteries since the presence of a variety of defects with different dimensions may promote ion diffusion and provide extra storage sites. However, manipulating defects and obtaining an in-depth understanding of their role in electrode materials remain challenging. Here, we deliberately introduce a considerable number of twin boundaries into spinel cathodes by adjusting the synthesis conditions. Through high-resolution scanning transmission electron microscopy and neutron diffraction, the detailed structures of the twin boundary defects are clarified, and the formation of twin boundary defects is attributed to agminated lithium atoms occupying the Mn sites around the twin boundary. In combination with electrochemical experiments and first-principles calculations, we demonstrate that the presence of twin boundaries in the spinel cathode enables fast lithium-ion diffusion, leading to excellent fast charging performance, namely, 75% and 58% capacity retention at 5 C and 10 C, respectively. These findings demonstrate a simple and effective approach for fabricating fast-charging cathodes through the use of defect engineering.

## Abstract (web summary)

Defect modulation and understanding its impact on electrode materials remain challenging. Here, authors introduce twin boundaries into spinel cathode and demonstrate that their presence enables fast lithium ion diffusion.

## Introduction

Crystallographic defects, such as vacancies, antisites, and dislocations, restrict the physical and chemical properties of crystalline materials and even play a decisive role in determining their various performances^1–4^. Generally, defects can be grouped into three types on the basis of their dimensionality, i.e., point defects, linear defects, and planar defects. Point defects usually emerge in the form of vacancies, substitutional, or interstitial atoms^5–7^, while linear and planar defects are mostly exhibited in the forms of dislocations and boundaries, respectively^8,9^. Although the concentration and distribution of various defects can be investigated and determined accurately by taking advantage of advanced analysis techniques such as high-resolution transmission electron microscopy and neutron diffraction methods, controlling and manipulating the concentration and distribution of various defects in the lattice of given materials is still nontrivial and challenging in practice.

Regarding electrode materials for lithium batteries, crystallographic defects are undoubtedly an important factor that seriously affects electrochemical performance. Different types of defects can exert different influences on the electrochemical performance of electrode materials. Point defects represented by vacancies (such as oxygen vacancies and transition metal vacancies in both cathode and anode materials^10–12^), atomic exchanges or so-called antisite defects (e.g., Li–Fe exchange in LiFePO_4_ and Li–Ni exchange in LiNiO_2_^13,14^) and substitutional atoms (e.g., Al^3+^ and Mg^2+^ doping in LiCoO_2_^15,16^) can be relatively easier to control by varying the composition and synthesis process to optimize the electrochemical performance of electrode materials^17,18^. In contrast to point defects, the distribution of planar defects is less homogeneous and depends largely on the variation in their thermodynamic state. Therefore, there are only a limited number of studies that explore the phenomenon and mechanism of planar defects as well as effective approaches to control them in electrode materials^8,19^. For instance, Moriwake et al.^20^ found that the cathode voltage decreased by 0.2 V in the vicinity of a coherent twin boundary relative to the perfect crystal in LiCoO_2_ through first-principles calculations, and Nie et al.^21^ found that Li ions preferred to intercalate in the vicinity of the grain boundary, which acted as a conduit for Li-ion diffusion in SnO_2_ nanowires and promoted the possibility of these grain boundaries as efficient lithium pathways. As an important type of defect, the existence of planar defects will inevitably influence ionic diffusion in electrode materials for lithium batteries. Therefore, further research and development are required to create and control planar defects during the synthesis procedure of electrode materials, which is of great significance not only for improving their performance but also for understanding the relationship between defects and electrochemical properties in electrode materials.

At present, layered ternary cathode materials (LiNi_*x*_Co_*y*_Mn_*z*_O_2_, *x* + *y* + *z* = 1), lithium iron phosphate (LiFePO_4_) and spinel lithium manganese cathode materials (LiMn_2_O_4_) are mainstream commercial cathode materials in lithium batteries used for rechargeable energy storage^22,23^. Among them, spinel LiMn_2_O_4_ is widely used in large-scale energy storage and electric vehicles owing to its advantages of low cost, nontoxicity, abundant reserves, and a certain degree of competitiveness in many aspects. However, the dissolution of manganese in the electrolyte^24^, the Jahn–Teller distortion of Mn^3+^ and the poor rate performance of LiMn_2_O_4_ limit its wide application in industry^25,26^. To date, research focused on the modification of LiMn_2_O_4_ has mainly been performed by elementary doping^27,28^, interfacial modification^29^, and nanoprocessing^30^ to improve conductivity and protect the interface, thereby improving the rate performance and cycling stability of materials. Given that the introduction of point or planar defects as discontinuities in lattices can be a reliable and effective approach to optimize the structure and properties of LiMn_2_O_4_, it is worthwhile to further optimize the electrochemical performance of LiMn_2_O_4_ through defect engineering and to obtain an in-depth understanding of the mechanism of defects on cathodes, which is beneficial to further promote the performance of full batteries.

Herein, we successfully create and introduce a considerable number of twin boundary defects into the lattice of spinel lithium manganate oxide materials via a defect engineering approach to enable fast lithium-ion diffusion for fast-charging batteries. By applying advanced structural characterization methods, including neutron diffraction and atomic-resolution scanning transmission electron microscopy (STEM), the morphology and distribution of these defects, in particular planar defects, are revealed at the atomic level. Based on the experimental results, we carried out simulation calculations to elaborate the structure–function relationship of this material with defects and found that the fast-charging mechanism is associated with the atomic arrangement near the twin boundaries. We open up the new concept of introducing twin boundary planar defect engineering to enhance lithium-ion diffusion for fabricating fast-charging cathode materials that can be applied in power lithium-ion batteries.

## Results

### Synthesis and characterization

**Fig. 1:**
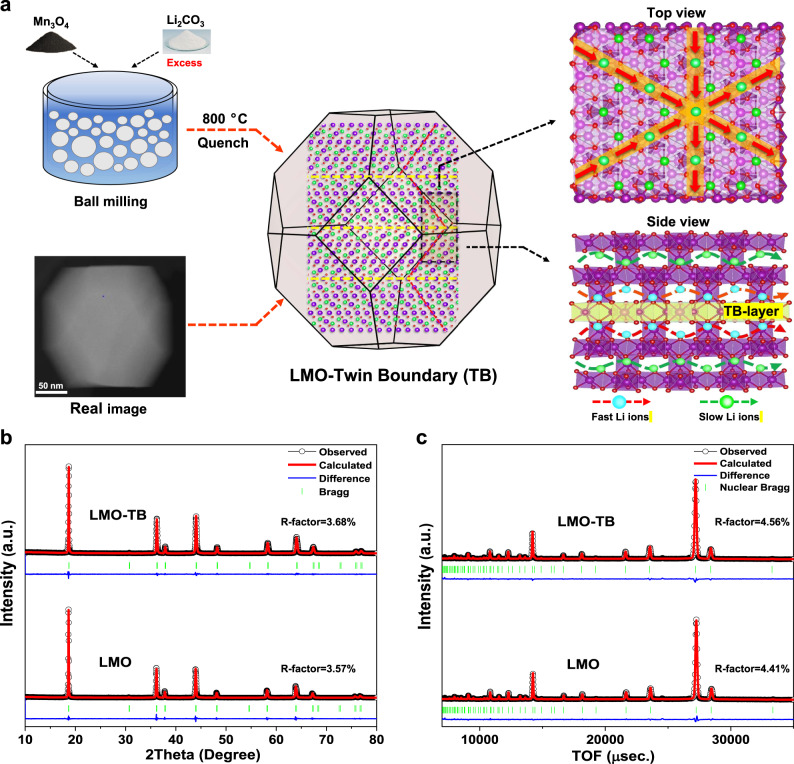
Schematic illustration of the synthesized LMO-TB and structural characterization. **a** Schematic of the fast Li-ion migration channels in LMO-TB. The blue, green, red, and purple balls represent fast lithium ions (Li), slow lithium ions (Li), oxygen (O), and manganese (Mn) atoms, respectively. **b** XRD and **c** neutron powder diffraction refinement patterns for LMO-TB and pristine LMO. Scale bar: 50 nm (**a**).

The spinel lithium manganate oxide cathode with a considerable number of twin boundaries (LMO-TB) was synthesized by adding excess lithium and adjusting the sintering conditions. The fabrication process is schematically shown in Fig. 1a (see the Methods section for details). In brief, commercialized Li_2_CO_3_ and Mn_3_O_4_ (molar ratio of Li:Mn = 1.08:2) were mixed with ball milling and then calcined at 800 °C in air for 15 h followed by a quenching process to form spinel LMO-TB microcrystals (2Mn_3_O_4_ + 1.5Li_2_CO_3_ → 3LiMn_2_O_4_ + 1.5CO_2_↑)^31^. In addition, pristine LMO (Li:Mn =  1:2) was synthesized with the same parameters and used as a reference. The addition of excess lithium salt is the decisive factor for the formation of a twin boundary defect structure in LMO-TB, which regulates lithium diffusion in the spinel crystal structure (Fig. 1a).

The crystal structures of LMO-TB and LMO were characterized by X-ray diffraction and neutron diffraction, as shown in Fig. 1b, c. All the diffraction peaks of the LMO-TB and LMO samples can be indexed in the spinel structure with space group *Fd-3m*. No heterogeneous phase is detected in either sample, and the sharp Bragg peaks indicate the good crystallinity of both samples.

Accurate structural information was obtained by combined refinement using both X-ray and neutron diffraction patterns, and the results are listed in Supplementary Tables 1–3. According to the refinement results, it is evident that the proportion between Li and Mn in pristine LMO is consistent with the target product, suggesting the formation of stoichiometric LiMn_2_O_4_ (see Supplementary Table 1). Once the content of the precursor lithium source is increased in LMO-TB, a small amount of Li–Mn exchange is detected, and the elemental ratio between Li and Mn increases to 1.051:1.949 (Supplementary Table 2). The lattice parameters of the two materials are shown in Supplementary Table 3, and only a subtle decrease (~0.04%) can be recognized in LMO-TB. Moreover, the elemental ratios between Li and Mn measured by inductively coupled plasma-atomic emission spectroscopy (Supplementary Table 4) are also consistent with the joint refinement results.

**Fig. 2:**
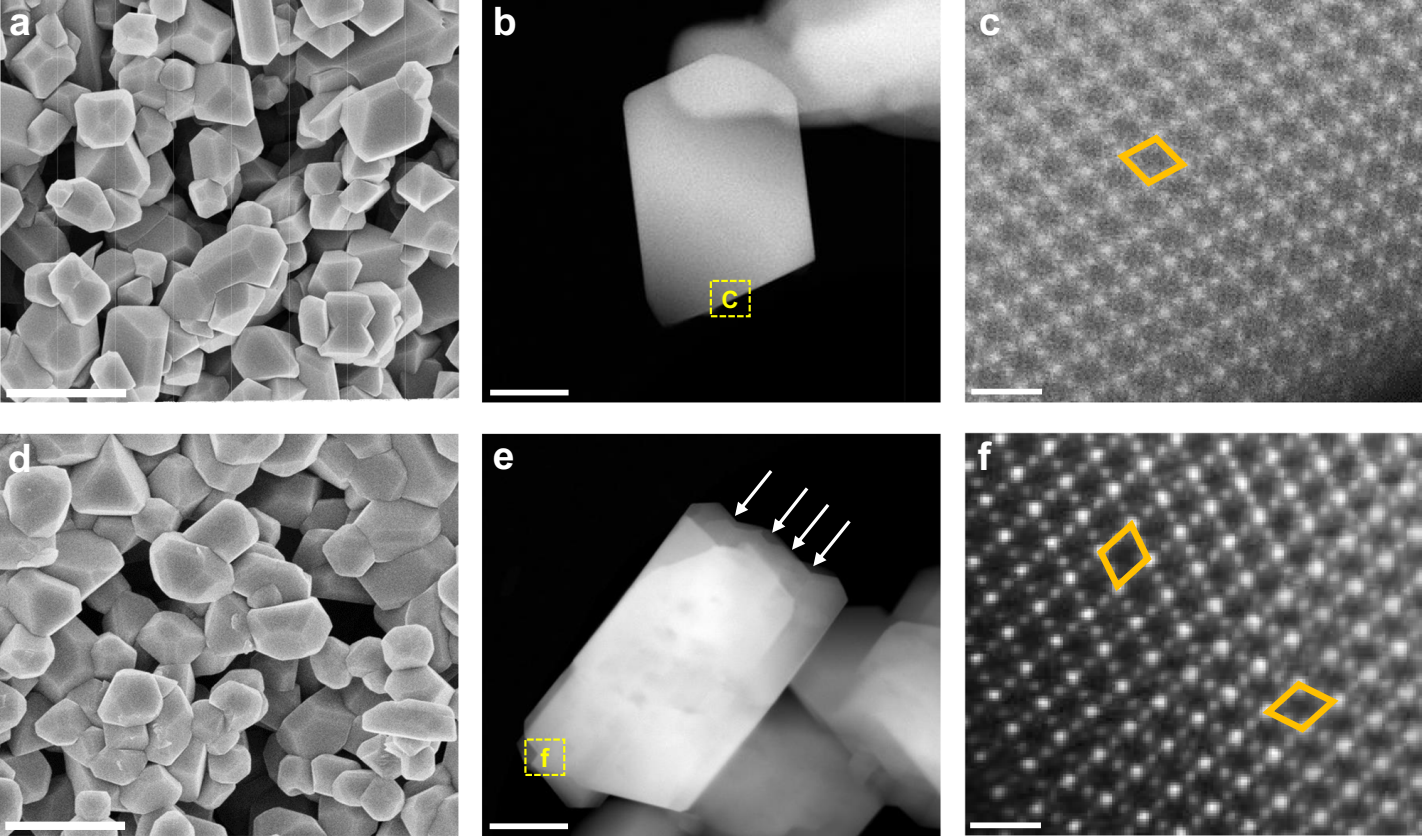
Structural characterizations of LMO and LMO-TB. **a, d** SEM, **b, e** high-magnification HAADF STEM and **c, f** atomically resolved HAADF STEM images of **b** LMO and **e** LMO-TB. Only Mn atomic columns are visible, which are arranged in a rhombus shape. Scale bars, 500 nm (**a**, **d**); 100 nm (**b**, **e**); 0.5 nm (**c**, **f**).

Furthermore, the impact of excess lithium salts on the LMO-TB sample was clarified by scanning electron microscopy (SEM), scanning transmission electron microscopy (STEM), and X-ray photoelectron spectroscopy (XPS). The SEM images (Fig. 2a, d) demonstrate that both LMO and LMO-TB are well crystallized as microcrystals, exhibiting regular polyhedral shapes and possessing very similar particle size distributions, as illustrated in Supplementary Fig. 1. The STEM images of the selected microcrystals of these two samples also show similar polyhedral morphology, and the corresponding EDS elemental maps of Mn and O reveal a uniform elemental distribution (Supplementary Fig. 2). The binding energies of Mn 2*p*_1/2_ and 2*p*_3/2_ in the LMO and LMO-TB samples (Supplementary Fig. 3) are both approximately 653.3 eV and 641.2 eV, respectively, which are consistent with previous reports^32,33^. The Mn 2*p* peaks for LMO and LMO-TB were fitted with the 2*p*_1/2_ and 2*p*_3/2_ peaks of Mn^4+^ and Mn^3+^, and the results show that 49.5% Mn^4+^ and 50.5% Mn^3+^ are present in LMO, while 55.4% Mn^4+^ and 44.6% Mn^3+^ are present in LMO-TB. This result indicates that when excess lithium ions are introduced to the bulk, more Mn^4+^ ions are required to equilibrate the valence in LMO-TB.

To explore the microstructures, both samples were further characterized by spherical aberration-corrected STEM measurements, as shown in Fig. 2b, c and Fig. 2e, f. All images were recorded along the <110> direction. The atomic structure of pristine LMO was first surveyed. High-angle annular dark-field (HAADF) STEM images from randomly selected locations of LMO are presented in Fig. 2b, c at gradually increasing magnifications. Particularly, Fig. 2c shows the atomic-resolution image, in which all the white dots represent atomic Mn columns neatly arranged in a rhombus. Similarly, the HAADF STEM images of the selected region of an LMO-TB microcrystal at different magnifications are shown in Fig. 2e, f, respectively. As illustrated in Fig. 2f, one of the prominent features in the LMO-TB lattice is the occurrence of twinning structures accompanied by a considerable number of twin boundaries (TB). This can be evidenced by the rotation of the Mn rhombus in the upper and lower twin variants.

To prove that the twinning structure is an intrinsic feature in LMO-TB, whereas it is not a common feature in LMO, the amount of data was increased during the STEM measurements by surveying different positions of different microcrystals for both the LMO and LMO-TB samples (see Supplementary Figs. 4 and 5). The results show that twinning structures can only be observed in LMO-TB. Conventional bright-field TEM experiments were also performed to image the twin boundaries as well as the twin variants. Regarding the examined particles, the twin boundary can be clearly observed, and the twin boundary is generated throughout the interior of the particles (Supplementary Fig. 6). Moreover, we also performed electron backscattered diffraction (EBSD) experiments on the LMO-TB sample since EBSD is a complementary technique when exploring defects and surveying the orientation distribution of target materials^34^. Given that the twinning structure can be clearly observed from the cross-section of the LMO-TB particles via EBSD, as illustrated in Supplementary Figs. 7 and 8, it can be unambiguously concluded that the twin boundary in LMO-TB is generated throughout the interior of a particle.

**Fig. 3:**
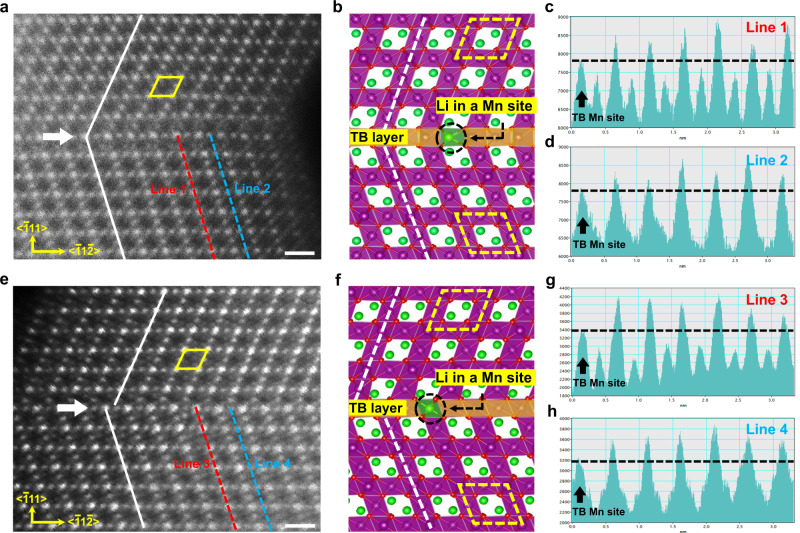
Twin boundaries in the LMO-TB cathode. **a**, **e** Atomically resolved HAADF STEM images and **b**, **f** structural schematic of the **a**, **b** symmetrical and **e**, **f** asymmetrical twin boundaries. The positions of the twin boundaries are indicated by white arrows (Li: green, Mn: purple, and O: red). **c**, **d** Corresponding atomic contrast curve of Line 1 and Line 2 in **a**. **g**, **h** Corresponding atomic contrast curve of Line 3 and Line 4 in **e**. Scale bars: 0.5 nm (**a**, **e**).

Closer examinations of the twinning structure in LMO-TB show that there are two types of twinning structures, i.e., symmetrical and asymmetrical twinning structures, as shown in Fig. 3. The local atomic structures near the twin boundaries are also different. Regarding the symmetrical twinning structure (Fig. 3a, b), the upper and lower twinning variants are connected via a pure mirror operation. The position of the {$\bar{1}$11} mirror plane (i.e., twin boundary) is indicated by the white arrow in Fig. 3a. The other {1$\bar{1}$1} planes for both twin variants are also marked by white solid lines to highlight the continuity of atomic layers crossing the boundary. Regarding the asymmetrical twinning structure (Fig. 3e, f), the two variants show an additional shift along the {$\bar{1}$11} mirror plane (displacement vector ***R*** = *a* < $\bar{1}$1$\bar{2}$>/8, where *a* ≈ 8.23 Å is lattice parameter of LMO-TB). As a consequence, the {1$\bar{1}$1} planes become staggered crossing the twin boundary (see white solid lines). It should be emphasized that according to our statistical measurement, asymmetrical boundaries are found to be more popular in the LMO-TB sample, as demonstrated in Supplementary Fig. 5.

Given that only LMO-TB contains an excess amount of lithium compared to pristine LMO (while the other growth parameters are identical), it is reasonable to deduce that the formation of twinning structures in LMO-TB is associated with the effect of excess lithium. To gain insight into this phenomenon, first-principles calculations were performed to understand the structural stability of LMO with and without excess lithium. According to the refinement results from XRD and NPD, the excess of lithium ions will substitute Mn ions (16*d* Wyckoff site). Based on this scenario, we calculated the formation energy of twin boundaries in LMO and LMO-TB, as shown in Supplementary Figs. 9 and 10, respectively. We conclude that with the introduction of excess lithium to LMO, the formation energy for symmetrical twin boundaries is reduced from 0.074 eV Å^−2^ to 0.070 eV Å^−2^, while the formation energy for asymmetrical twin boundaries is reduced from 0.014 eV Å^−2^ to 0.005 eV Å^−2^. This result means that the formation of twinning structures in LMO is energetically favorable. In particular, the asymmetrical twining structure possesses a lower formation energy; thus, it is prone to emerge during the sample synthesis process.

Within the framework of the spinel structure, Li atoms occupy the 8*a* Wyckoff site. In the <110> projection, the two atomic columns of Li (green) are located in the middle of the rhombus formed by atomic Mn columns (purple), as illustrated in Fig. 3b, f. To demonstrate the structural differences between the twin boundary region and bulk region in LMO-TB, the intensity change for the atomic Mn columns near the symmetrical and asymmetrical twin boundaries was studied. As shown in Fig. 3a, e, four line profiles are extracted from the symmetrical and asymmetrical twinning structures, where **Line 1** and **Line 3** (red dashed lines) represent the Mn atoms on one side of the rhombus, while **Line 2** and **Line 4** (blue dashed lines) represent the Mn atoms at the midpoint of each side of the rhombus. The atomic intensity curves of **Lines 1–4** can be seen in Fig. 3c, d, g, h, which all show that the intensities of Mn atoms located at the twin boundaries are weaker than those in the bulk region (see black dashed lines as an eye guide). Moreover, another 16 line profiles for symmetrical and asymmetrical twinning structures were also performed in Supplementary Figs. 11 and 12, showing the same results. This outcome means that excess lithium will mostly occupy the Mn sites at twinning boundaries, resulting in the decreased intensity of these columns (the atomic number of lithium is far less than that of manganese). This observation is also supported by the theoretical calculations in Supplementary Figs. 9 and 10, which predict that excess lithium occupying the Mn site at twin boundaries is the most stable structure. Therefore, the twinning boundaries in LMO-TB can accommodate more lithium.

Since we used HAADF STEM (so-called Z-contrast image), the Li atoms are not visible in the images. However, we did observe a signal at the projected 8*a* site close to the twin boundary of LMO-TB, as revealed by the white spots inside the rhombus (see the yellow rhombus in Supplementary Fig. 13a, e and the detailed analysis in Supplementary Fig. 13). This observation means that a moderate number of Mn atoms migrate to the 8*d* Wyckoff positions, which are originally occupied by Li atoms near the twin boundary. In contrast, white spots are not detected inside the rhombus in the lattice away from the twin boundary region. Based on first-principles calculations, we determine the formation energies of Li–Mn exchange in the two different circumstances, i.e., near the twin boundary region and away from the twin boundary region. The results show that the formation energy for Li-Mn exchange near the twin boundary is 0.49 eV, which is much lower than that of 0.60 eV in the bulk region, as shown in Supplementary Fig. 14. Therefore, it is concluded that the Li–Mn exchange prefers to distribute around twin boundaries, due to a lower formation energy.

### Electrochemical performance

**Fig. 4:**
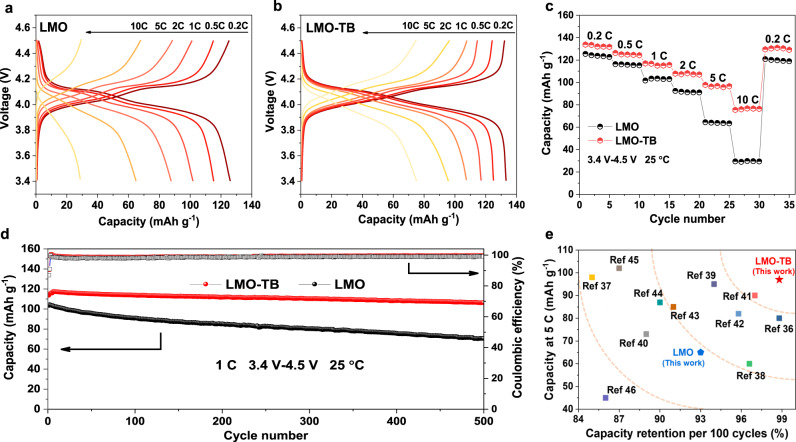
Electrochemical properties of the cells fabricated with LMO and LMO-TB cathodes. **a**, **b** Charge-discharge curves, **c** rate capacities, **d** long-term cycling, and **e** electrochemical performance compared with reported LMO cathode materials. The detailed cell properties and cycling conditions for the different cells investigated in this comparison are listed in Supplementary Table 5.

To evaluate the electrochemical properties of LMO-TB materials, the as-synthesized electrodes were initially subjected to a varying current rate test in a potential window of 3.4–4.5 V at 25 °C. To verify and highlight the cooperative functions of twin boundaries in the crystals, the conventional LMO cathode without twin boundary defects was also measured under the same conditions for comparison. First, two electrodes were activated at a low rate of 0.1 C and the initial charge–discharge (Supplementary Fig. 15) showed that the initial discharge capacities of LMO-TB and LMO were 138 mAh g^−1^ and 133 mAh g^−1^, respectively, with coulombic efficiencies of 91% and 85%, respectively. The subsequent galvanostatic charge–discharge profiles of the two samples at different rates of 0.2, 0.5, 1.0, 2.0, 5.0, and 10.0C are depicted in Fig. 4a, b. Our synthesized LMO cathode, like most reported LMOs, shows poor rate performance. The capacities of LMO are deduced to be 128, 114, 101, 87, 65, and 30 mAh g^−1^ at rates of 0.2, 0.5, 1, 2, 5, and 10C, respectively. However, LMO-TB exhibits superior rate performance. The capacities of LMO-TB are 132, 127, 118, 102, 98, and 78  mAh g^−1^ at rates of 0.2, 0.5, 1, 2, 5, and 10C, respectively, which are always higher than the respective values of the LMO sample (Fig. 4c). Specifically, the capacity retentions of LMO-TB at high rates of 5 and 10 C are up to 75% and 58%, respectively, demonstrating its excellent fast-charging performance, which is suitable for power lithium-ion batteries. In addition, the cycling stability of the two samples was also tested at a high rate of 1C. The capacities of the LMO-TB and LMO samples are 107 mAh  g^−1^ and 71 mAh g^−1^, respectively, after 500 cycles, corresponding to retention rates of 94% and 69%, respectively (Fig. 4d). The excellent capacity retention of LMO-TB can be attributed to the increased Mn^4+^/Mn^3+^ ratio as well as the occurrence of the Li–Mn exchange in LMO-TB, which is associated with excess lithium. The Li–Mn exchange can suppress the generation of irreversible phase transitions, stabilize the structure and thereby inhibit the Jahn–Teller effect induced by Mn^3+^ during the charge and discharge process^26,35^. Furthermore, after comparing the high-rate (5C) capacity and retention rate of LMO-TB with reported LMO cathode materials^25,36–45^, clearly, our LMO-TB is located at the top right corner in Fig. 4e, demonstrating that it is the state-of-the-art LMO cathode for fast-charging lithium-ion batteries.

**Fig. 5:**
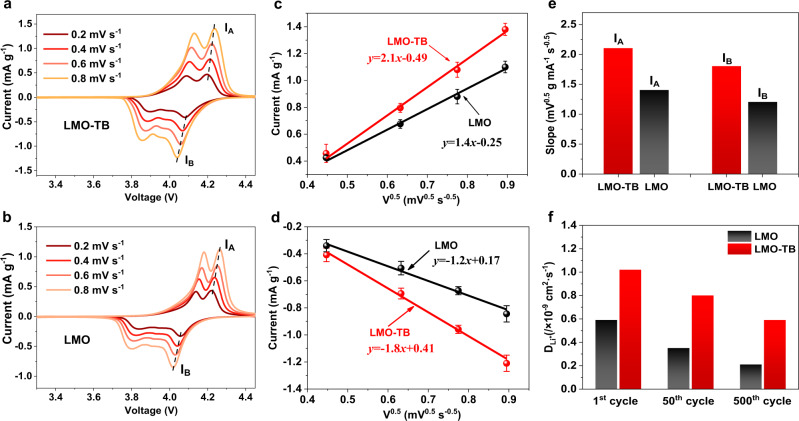
Lithium-ion diffusion rate of the LMO and LMO-TB cathodes. **a**, **b** CV curves at different rates, **c**, **d** linear relationship between the major **c** oxidation (*I*_A_) and **d** reduction (*I*_B_) peaks and scan rates. Error bars represent the standard deviations of the value for oxidation and reduction peaks in CV curves, **e** compared slope values of *I*_A_ and *I*_B_, **f** lithium-ion diffusion coefficients at different cycle numbers.

To clarify the cause of the excellent high power performance of spinel cathodes, cyclic voltammetry (CV) at different scan rates was carried out to investigate the Li-ion diffusion rate in the two cathodes (Fig. 5a, b). Major peaks (*I*_A_) and (*I*_B_) can be observed in the charge and discharge processes, respectively, with *I*_A_ representing the Mn-ion oxidation process and *I*_B_ representing the Mn-ion reduction process. The lithium-ion diffusion kinetics can thus be obtained according to the Randles-Sevcik equation^46,47^,

1 $${{{I}}}_{{\rm{P}}}=(2.65\times {10}^{5}){n}^{1.5}S{D}_{{\rm{Li}}}^{,0.5}{C}_{{\rm{Li}}}{\upnu }^{0.5}$$

where *I*_P_ is the peak current, *n* represents the electron number, *S* is the electrode area, *D*_Li_ is the Li-ion diffusion coefficient, *C*_Li_ is the Li-ion concentration in the electrochemical reaction, and *ν* is the scan rate. As shown in equation (1), *D*_Li_ has a positive correlation with the slopes of the *I*_P_/*ν*^0.5^ curves (Fig. 5c, d). According to the change in peak currents *I*_A_ and *I*_B_ at different scan rates of 0.2, 0.4, 0.6, 0.8 mV s^−1^, the slopes of *I*_P_/*ν*^0.5^ curves are fitted in Fig. 5e, and the results show that both slopes of *I*_A_/*ν*^0.5^ and *I*_B_/*ν*^0.5^ of LMO-TB are higher than LMO, indicating that the Li-ion diffusion rate in LMO-TB is notably faster than LMO.

Furthermore, the electrochemical impedances of the two materials at the same discharge stage of 4V were also tested, and the impedance spectra at three different cycles, i.e., the first cycle (Supplementary Fig. 16a), 50th cycle (Supplementary Fig. 16b) and 500th cycle (Supplementary Fig. 16c) were obtained. All impedance spectra are composed of a high-frequency semicircle that corresponds to *R*_ct_ (interfacial charge transfer resistance) and a low-frequency tail that is associated with the Li^+^ diffusion process^48,49^. Supplementary Figure 16 shows that the *R*_ct_ of LMO nearly triples throughout the whole process with an increasing number of cycles, while the *R*_ct_ of LMO-TB only slightly increases. Additionally, the impedance at low frequency of three different cycles was fitted to obtain the corresponding lithium-ion diffusion rate, as shown in Fig. 5f. The details of the fitting process can be found in the Supporting Information and Supplementary Fig. 17. On the basis of the fitting results, the lithium-ion diffusion rates of LMO-TB at the 1st, 50th, and 500th cycles are deduced to be 1.02 × 10^−9^, 0.83 × 10^−9^, and 0.66 × 10^−9^ cm^2^ s^−1^, respectively, which are higher than the lithium-ion diffusion rate of LMO in any cycles. In particular, the lithium-ion diffusion rate in LMO-TB is ~10^2^ times that of reported layered ternary oxides (~10^−11^ cm^2^ s^−1^)^50,51^ and 10^5^ times that of LFP phosphate materials (~10^−14^ cm^2^ s^−1^)^52,53^, demonstrating the great kinetics advantages of the LMO-TB cathodes applied in the prepared fast-charging lithium-ion batteries.

## Discussion

**Fig. 6:**
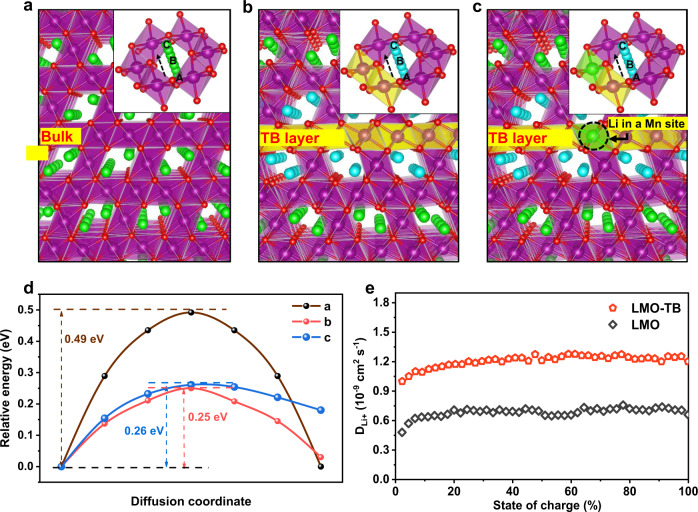
Fast lithium-ion diffusion mechanism of LMO-TB. **a**–**c** Schematic diagram of lithium-ion diffusion in the **a** bulk structure and at the **b** asymmetrical twin boundary and **c** asymmetrical twin boundary with excess lithium. The insets of **a**–**c** are the tetrahedron-octahedron-tetrahedron diffusion tunnels. **d** Energy barrier for lithium-ion diffusion under the three conditions of **a**–**c**. **e** Lithium-ion diffusion rates of LMO and LMO-TB, as calculated by GITT. The purple and yellow octahedra represent Mn–O octahedrons in bulk and at the twin boundary plane, respectively. The blue octahedron represents the Li-O octahedron in the twin boundary plane, and the lithium ions at the 8a Wyckoff site transfer from inside to outside along the channel.

The markedly improved lithium diffusion kinetics and fast-charging performance of the LMO-TB cathode, compared with conventional LMO, is found to be closely associated with twin boundary defect structures in the crystal. We simulated the diffusion processes in different situations, i.e., lithium ions diffusing in the defect-free bulk (Fig. 6a), along the asymmetrical twin boundary (Fig. 6b) and along the asymmetrical twin boundary with excess lithium (Fig. 6c). The local structures for a tetrahedron-octahedron-tetrahedron tunnel of lithium ions in LMO and LMO-TB are illustrated in the inset graph of Fig. 6a–c, in which the yellow and blue octahedrons represent Mn–O and Li-O octahedra in the twin boundary plane, respectively. In both situations, the Li ion jumps from a tetrahedral site to the adjacent tetrahedral site through an octahedral vacancy surrounded by six Mn ions in the octahedral site. This channel is perpendicular to the {111} surface. The energy barrier of this channel is mainly influenced by the six closed Mn ions around the octahedral vacancy^54–56^. The calculated results are shown in Fig. 6d, which demonstrate that the energy barriers of Li-atom diffusion along the asymmetrical twin boundary and the asymmetrical twin boundary with excess lithium are just 0.25 eV and 0.26 eV, respectively. These values are much lower than 0.49 eV in the defect-free bulk region. Additionally, the energy barrier of lithium-atom diffusion along the symmetrical twin boundary (see Supplementary Fig. 18) is lower than that in the defect-free bulk region, proving that lithium-ion diffusion across the twin boundary is more feasible and facile.

It is worth emphasizing that according to the Arrhenius equation, i.e. $k=A{e}^{-{E}_{a}/{RT}}$, a decrease in the energy barrier of 0.23 eV, i.e., the energy difference between *E*_a_ in the defect-free region and *E*_a_ in the twin boundary region, can increase the ionic conductivity by four orders of magnitude. Knowing that most of the inorganic solid electrolytes for Li-ion batteries have an *E*_a_ of ~0.25 eV, the twin boundaries in LMO-TB can indeed be regarded as “inborn” electrolytes that infiltrate into the bulk phase. They play the role of fast diffusion lanes for Li ions; thus, we can expect there to be a sizeable number of Li ions that can travel along these lanes and reach the core of the particle in a much shorter time than pristine LMO. The proposed diffusion process of lithium ions in LMO-TB is proposed and displayed in Supplementary Figs. 19 and 20. Based on the calculation results, we can conclude that the twin boundary can provide an expressway for lithium diffusion by effectively decreasing the energy barrier for lithium ions.

To further verify the calculation results, the Galvanostatic Intermittent Titration Technique (GITT) test was performed. The GITT curves of both samples were obtained at a rate of 0.05C (Supplementary Fig. 21)^57^. Furthermore, the lithium-ion diffusion rate at different charging states was calculated from the GITT test and is shown in Fig. 6e^58,59^. It is evident that the lithium-ion diffusion rate of the LMO-TB sample is higher than that of the LMO sample, which is also consistent with the result obtained by fitting the impedance test results. Moreover, a systematic study on the electrochemical performances and structural properties of spinel cathode materials with different contents of excess Li further validates the positive correlations between the kinetics of lithium-ion diffusion and the contents of defects in spinel LMO material, as demonstrated in Supplementary Figs. 22–25. It is also worth noting that both LMO and LMO-TB particles adopt mostly truncated octahedral shapes with an average particle size of a few hundred nanometers (see Supplementary Fig. 1); thus, the potential modification of the particle morphology and exposed facets associated with the formation of twin boundaries are expected to exert negligible influence on lithium-ion diffusion kinetics, which is different from the situation in other reports where tremendous changes in the morphology and particle size of spinel cathodes are designed^60^. Therefore, by combining the experimental results and calculations, we argue that the existence of twin boundaries lowers the barrier of the lithium-ion diffusion process and improves the lithium-ion diffusion rate. As a consequence, better fast-charging performance can be obtained.

In conclusion, by adding an appropriate excess of lithium during the synthesis of LMO cathode materials, a considerable number of twin boundaries were induced to form in the LMO materials. The existence of both symmetrical and asymmetrical twin boundaries were observed and identified by STEM and EBSD techniques. Combining experimental evidence and first-principle calculations, we conclude that the twin boundaries effectively promote the lithium-ion diffusion rate and result in the excellent electrochemical properties of LMO-TB with a capacity retention rate of 94% after 500 cycles and a capacity of 78 mAh g^−1^ at a rate of 10C. Our work demonstrates an effective strategy for improving the electrochemical performance of spinel cathode materials by a defect engineering approach, and it is believed to shed light on the role of planar defects and on designing new cathode materials for fast-charging lithium batteries.

## Methods

### Material synthesis

The pristine LiMn_2_O_4_ (LMO) sample was first synthesized by a solid-state synthesis method. Mn_3_O_4_ and Li_2_CO_3_ were mixed at a molar ratio of Li:Mn = 1:2 and then fully ground and stirred in a mortar. Subsequently, the mixtures were calcined in a muffle furnace at 500 °C for 5 h and then cooled to room temperature. Finally, the presintered products were pressed into disks and calcined at 800 °C for 15 h followed by quenching to room temperature in air. The LiMn_2_O_4_ with twin boundaries (LMO-TB) sample was synthesized following the same procedure, except a different molar ratio of Li:Mn = 1.08:2 was used.

### Structural characterization

The crystallographic structures of both samples (in the form of powders) were characterized by both X-ray and neutron diffraction measurements. X-ray diffraction (XRD) patterns were collected using a Bruker D8 DISCOVER diffractometer with a Cu Kα radiation source at room temperature. Neutron powder diffraction (NPD) experiments were performed on a time-of-flight (TOF) general purpose powder diffractometer (GPPD) at the China Spallation Neutron Source (CSNC). Cathode samples were loaded in 9.1 mm diameter vanadium cans, and neutron diffraction patterns were collected at room temperature with wavelength bands from 0.1 to 4.9 Å. The diffraction profiles were analyzed by using the Rietveld refinement method with the FULLPROF suite. Morphological images of the two samples were collected by scanning electron microscopy (SEM, ZEISS Supra-55). For the STEM investigations, both powder samples were first sonicated in ethanol for 15 min and then dropwise added by pipette onto lacy carbon-coated Cu multigrids. High-angle annular dark-field (HAADF) STEM imaging was carried out on an FEI Titan G2 ChemiSTEM 80−200 transmission electron microscope equipped with a high-brightness field-emission gun and a probe spherical aberration (*C*_S_) correction system. The microscope was operated at 200 kV. The convergence semiangle for HAADF imaging was ~25 mrad, while the collection semiangle was ~70–200 mrad. The valence states of Mn were measured by X-ray photoelectron spectroscopy (XPS) on an ESCA Lab220I-XL monochromatic Al Kα X-ray source. All the binding energies were calibrated with the C 1s signal at 284.8 eV. Electron backscattered diffraction (EBSD) images were obtained by an FEI Scios focused ion beam (FIB) equipped with an Oxford Symmetry EBSD detector. EBSD data were processed with the Oxford AZtec program and HKL CHANNEL 5 software.

### Electrochemical measurements

Electrochemical properties were evaluated using coin cells (CR2032) assembled in an argon-filled glove box (with H_2_O < 0.1 ppm. and O_2_ < 0.1 ppm.). LMO or LMO-TB, Super-P, and binder (polyvinylidene fluoride, PVDF) at a mass ratio of 8:1:1 was dissolved in *N*-methyl-2-pyrrolidone (NMP) to prepare a cathode slurry that was then cast onto Al foil and dried in a vacuum oven for 10 h at 100 °C to obtain the cathode electrode. Lithium metal foil (99.9%, Alfa Aesar) was used as the anode. Additionally, 1 M LiPF_6_ in a mixture of ethylene carbonate (EC), dimethyl carbonate (DMC), and diethyl carbonate (DEC) at a ratio of 1:1:1 (vol%) was used as the electrolyte. Celgard 2500 was used as the separator. To eliminate the interference of electrode properties on the battery performance, we fabricated two electrodes, i.e., LMO and LMO-TB electrodes, with almost identical coating thicknesses (~83 µm), mass loadings (~3.46 g/cm^2^), and porosities. Galvanostatic charge/discharge testing was performed by using a Neware Test System between 3.4 V and 4.5 V at different C-rates (0.2C–10C, 1C = 148 mA g^−1^). Electrochemical impedance spectroscopy (EIS) measurements were carried out on the cathodes at the discharged state of 3.4 V at frequencies from 10 mHz^−1^ MHz with a sinusoidal excitation voltage of 10 mV. The galvanostatic intermittent titration technique (GITT) was measured by a Moccor test cabinet.

### DFT calculations

All calculations were performed using the plane-wave projector-augmented wave method with an energy cutoff of 520 eV, as implemented in the Vienna ab initio simulation package (VASP). The Perdew-Burke-Ernzerhof (PBE) form of the generalized gradient approximation (GGA) was chosen as the exchange-correlation potential. The PBE + U approach was employed to take into account the short on-site Coulomb interaction (U) presented in the localized 3d electrons of Mn, with the U values set to 3.9 eV, as reported in the literature value. The structures were relaxed until the forces were less than 0.01 eV/Å, and the energy convergent standard was 10^−4^ eV. To simulate the bulk and grain boundaries of LiMn_2_O_4_, we built a conventional standard cell and a (1 × 1 × 3) supercell containing 168 ions. The Monkhorst-Pack mesh of the bulk and supercell was set to 3 × 3 × 3 and 1 × 2 × 1, respectively.

## Supplementary information

Supplementary Information

## Data Availability

The data supporting the findings of this study are available from the corresponding author upon reasonable request.
