# Supplementary material for: Twin boundary defect engineering improves lithium-ion diffusion for fast-charging spinel cathode materials
Source: Nat Commun. 2021 May 25;12:3085. doi: 10.1038/s41467-021-23375-7 (PMC8149699; doi:10.1038/s41467-021-23375-7)
Supplement: Supplementary file 1 — Supplementary Information [file 41467_2021_23375_MOESM1_ESM.pdf]

Supplementary Information

Twin Boundary Defect Engineering Improves Lithium-ion Diffusion for Fast-charging Spinel Cathode Materials

Authors:

Rui Wang[1,#], Xin Chen[1,#], Zhongyuan Huang[1,#], Jinlong Yang[2,#], Fusheng Liu[2], Mihai Chu[1], Tongchao Liu[1], Chaoqi Wang[1], Weiming Zhu[1], Shuankui Li[1], Shunning Li[1], Jiaxin Zheng[1], Jie Chen[3,4], Lunhua He[4,5,6]*, Lei Jin[7]*, Feng Pan[1]* and Yinguo Xiao[1]*

[1] School of Advanced Materials, Peking University, Shenzhen Graduate School, Shenzhen, 518055, China

[2] College of Materials Science and Engineering, Shenzhen University, Shenzhen, 518060, China

[3] Institute of High Energy Physics, Chinese Academy of Sciences, Beijing 100049, China

[4] Spallation Neutron Source Science Center, Dongguan 523803, China

[5] Beijing National Laboratory for Condensed Matter Physics, Institute of Physics, Chinese Academy of Sciences, Beijing 100190, China

[6] Songshan Lake Materials Laboratory, Dongguan 523808, China

[7] Ernst Ruska-Centre for Microscopy and Spectroscopy with Electrons, Forschungszentrum Jülich GmbH, 52425 Jülich, Germany

[#] These authors contributed equally: Rui Wang, Xin Chen, Zhongyuan Huang, Jinlong Yang

*email: lhhe@iphy.ac.cn; l.jin@fz-juelich.de; panfeng@pkusz.edu.cn; y.xiao@pku.edu.cn

Supplementary Tables

Supplementary Table 1: Joint refinement result of the neutron diffraction and XRD data of LMO.

	**Site**	**x**	**y**	**z**	**Biso (Å^2)**	**Occupancy**
**Li**	8*a*	0.1250	0.1250	0.1250	1.94(3)	0.999(3)
**Mn**	16*d*	0.5	0.5	0.5	1.23(3)	2.001(3)
**O**	32*e*	0.2626(1)	0.2626(1)	0.2626(1)	1.88(3)	4.000(3)

Supplementary Table 2: Joint refinement result of the neutron diffraction and XRD data of LMO-TB.

	**Site**	**x**	**y**	**z**	**Biso (Å^2)**	**Occupancy**
**Li1**	8*a*	0.1250	0.1250	0.1250	1.87(3)	0.975(3)
**Mn1**	8*a*	0.1250	0.1250	0.1250	1.87(5)	0.025(3)
**Li2**	16*d*	0.5	0.5	0.5	0.73(5)	0.076(3)
**Mn2**	16*d*	0.5	0.5	0.5	0.73(3)	1.924(3)
**O**	32*e*	0.2628(1)	0.2628(1)	0.2628(1)	1.60(3)	4.000(3)

Supplementary Table 3: Lattice parameters of LMO and LMO-TB.

**Samples**	***a*/*b*/*c* (Å)**	***V* (Å^3)**
**LMO**	8.2387(1)	559.2114(1)
**LMO-TB**	8.2353(1)	558.5194(1)

Supplementary Table 4: Elemental compositions of LMO and LMO-TB, as measured by ICP-AES.

**Samples**	**LMO**	**LMO-TB**
**Li**	1.010	1.066
**Mn**	2.016	1.985
**Li/Mn**	0.501	0.537

Supplementary Table 5: Cell properties and electrochemical performances of LMO in the references.

**Ref.**	Electrolyte	Loading (mg/cm^2), composition (LMO/carbon/ PVDF)	Cycling condition	Rate capacity (5C) mAh g^{-1}	Capacity retention per 100 cycles
**Ref [36]**	1 M LiPF6 EC/DMC/EMC (1:1:1)	3.5, 8/1/1	RT, 3.0-4.3 V	80	98.5%
**Ref [37]**	1 M LiPF6 in EC/DEC (1:1)	3.5, 8/1/1	RT, 3.0-4.5 V	97	85.0%
**Ref [38]**	1 M LiPF6 in PC (1:1)	1-2, 8/1/1	RT, 3.3-4.5 V	60	96.6%
**Ref [39]**	1 M LiPF6 EC/EMC (1:1)	\, 8/1/1	RT, 3.3-4.4 V	95	94.1%
**Ref [40]**	1 M LiPF6 EC/EMC (1:1)	3.5, 8/1/1	RT, 3.4-4.3 V	73	72.8%
**Ref [41]**	1 M LiPF6 in EC/DEC (1:1)	2, 8/1/1	RT, 3.0-4.4 V	90	97.0%
**Ref [42]**	1 M LiPF6 in EC/DEC (1:1)	2, 8/1/1	RT, 3.0-4.3 V	81	95.8%
**Ref [43]**	1 M LiPF6 in EC/DEC (1:1)	1.5, 8/1/1	RT, 3.0-4.5 V	85	91.0%
**Ref [44]**	1 M LiPF6 in EC/DMC (1:1)	\, 8/1/1	RT, 3.3-4.35 V	87	90.0%
**Ref [45]**	1 M LiPF6 EC/DMC/EMC (1:1:1)	\, 8/1/1	RT, 3.3-4.35 V	101	87.2%
**Ref [46]**	1 M LiPF6 in EC/DEC (1:1)	1.5, 8/1/1	RT, 3.0-4.3 V	45	86.0%
**LMO(this work)**	1 M LiPF6 EC/DMC/EMC (1:1:1)	3.45, 8/1/1	RT, 3.4-4.5 V	**65**	**93.5%**
**LMO-TB (this work)**	1 M LiPF6 EC/DMC/EMC (1:1:1)	3.45, 8/1/1	RT, 3.4-4.5 V	**98**	**98.8%**

Supplementary Figures

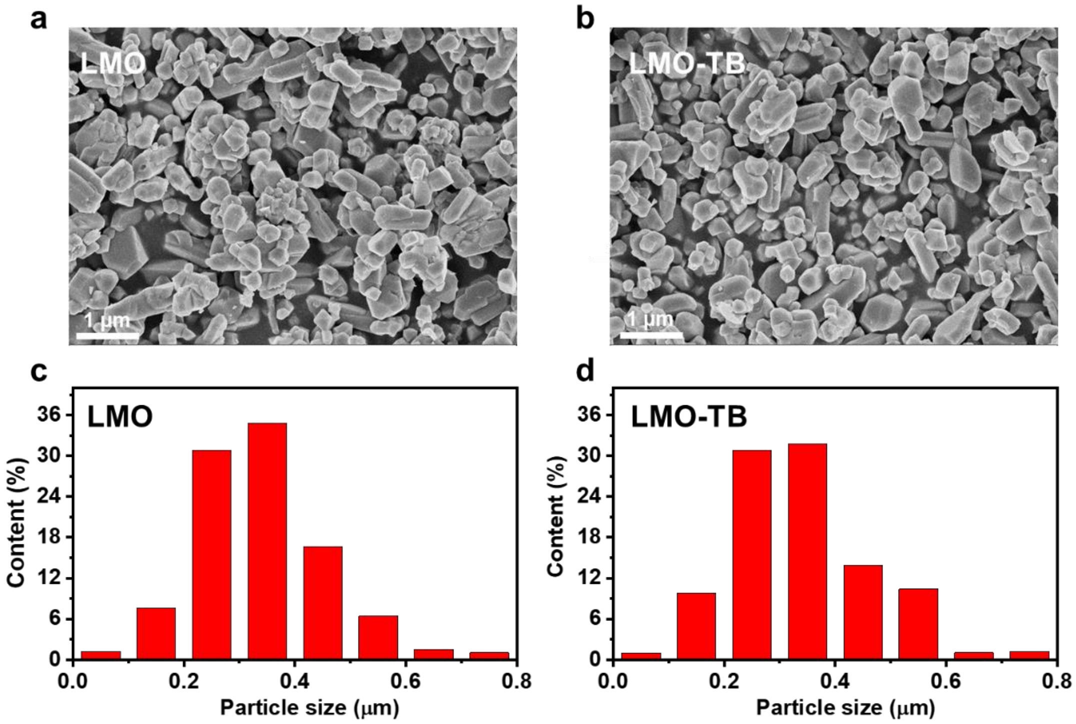

Supplementary Fig. 1: (a, b) SEM images and (c, d) size distributions of the (a, c) LMO and (b, d) LMO-TB particles.

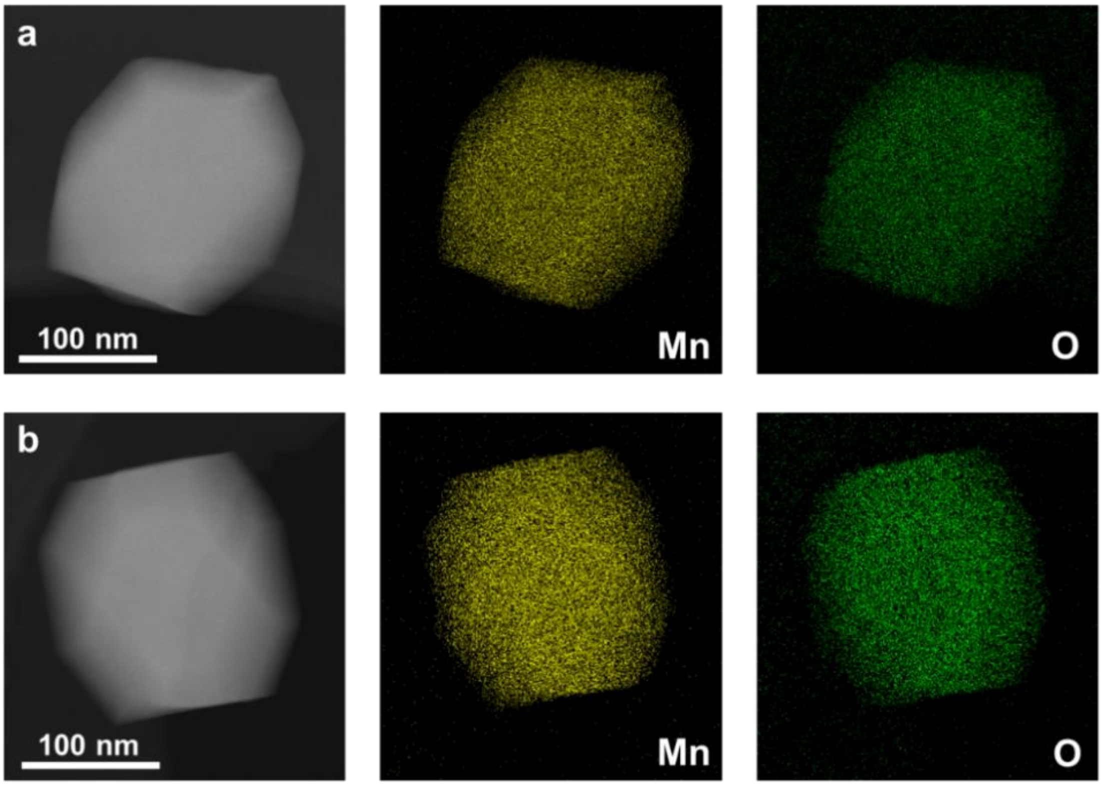

Supplementary Fig. 2: (a) STEM image of LMO and the corresponding energy dispersive X-ray spectroscopic elemental maps of Mn (yellow) and O (green). (b) STEM image of LMO-TB and the corresponding energy dispersive X-ray spectroscopic elemental maps of Mn (yellow) and O (green).

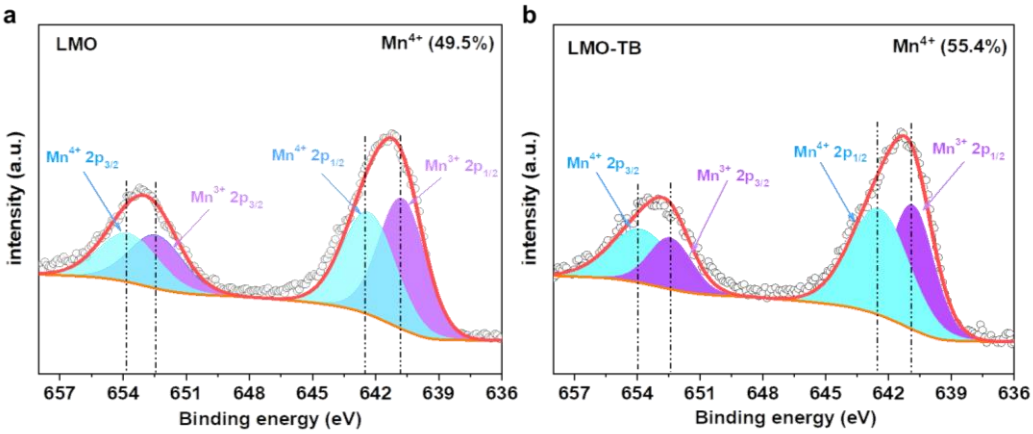

Supplementary Fig. 3: Mn $2p_{1/2}$ and $2p_{3/2}$ spectra and fitting results for the (a) LMO and (b) LMO-TB samples.

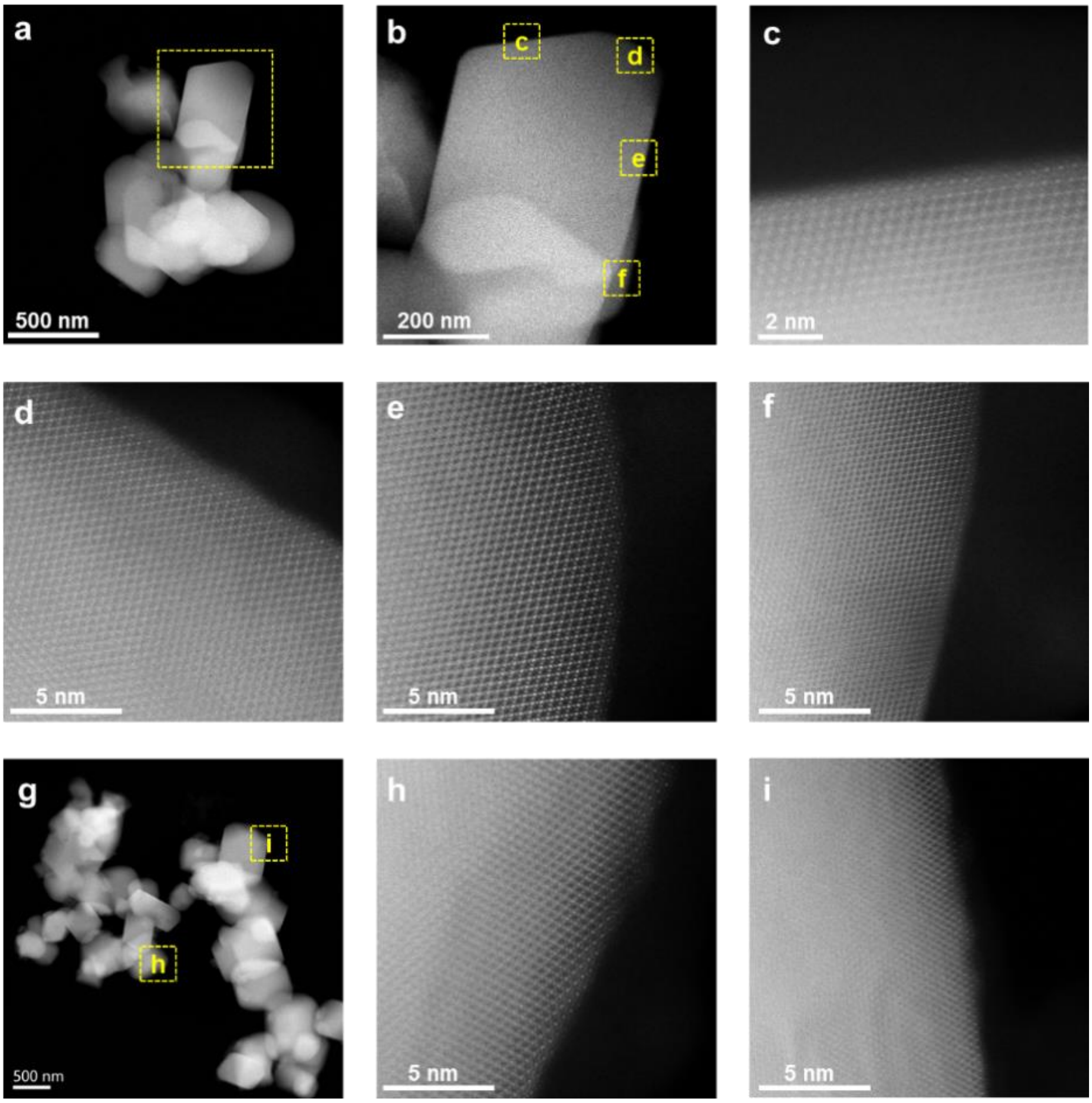

Supplementary Fig. 4: (a) Low-magnification HAADF STEM image of the LMO microcrystals, (b) Medium-magnification HAADF STEM image showing the selected area in (a). (c, d, e, f) Atomically resolved HAADF STEM images of the corresponding areas in (b). (g) Low-magnification, (h) and (i) atomic-resolution HAADF STEM images taken from other LMO microcrystals. The selected areas in (h) and (i) are indicated in (g).

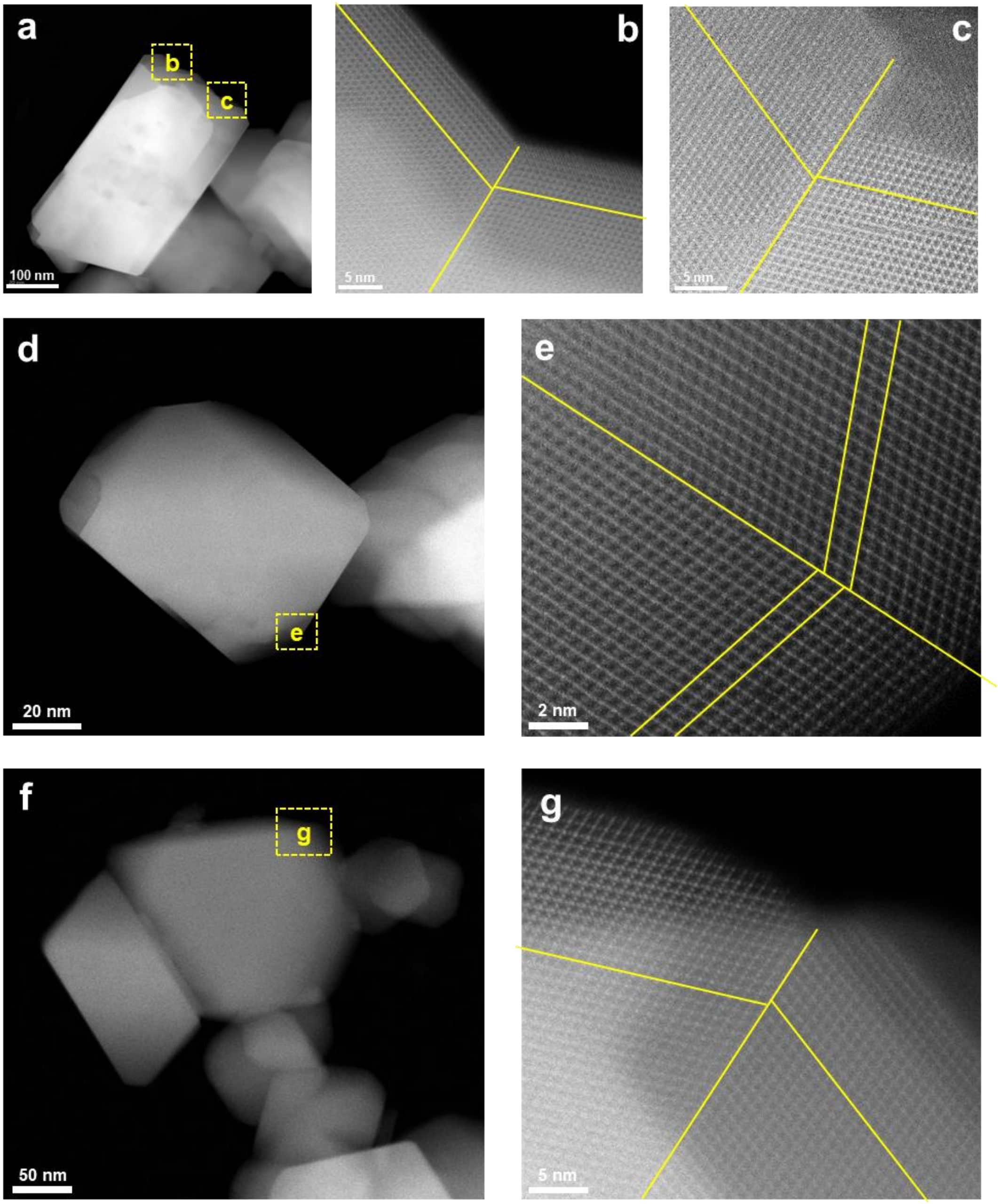

Supplementary Fig. 5: (a, d, f) Low-magnification HAADF STEM images showing three different LMO-TB microcrystals. (b, c, d, g) Magnified atomic-resolution HAADF STEM images from the areas marked by yellow frames in (a), (d) and (f), respectively, showing asymmetric twinning structures.

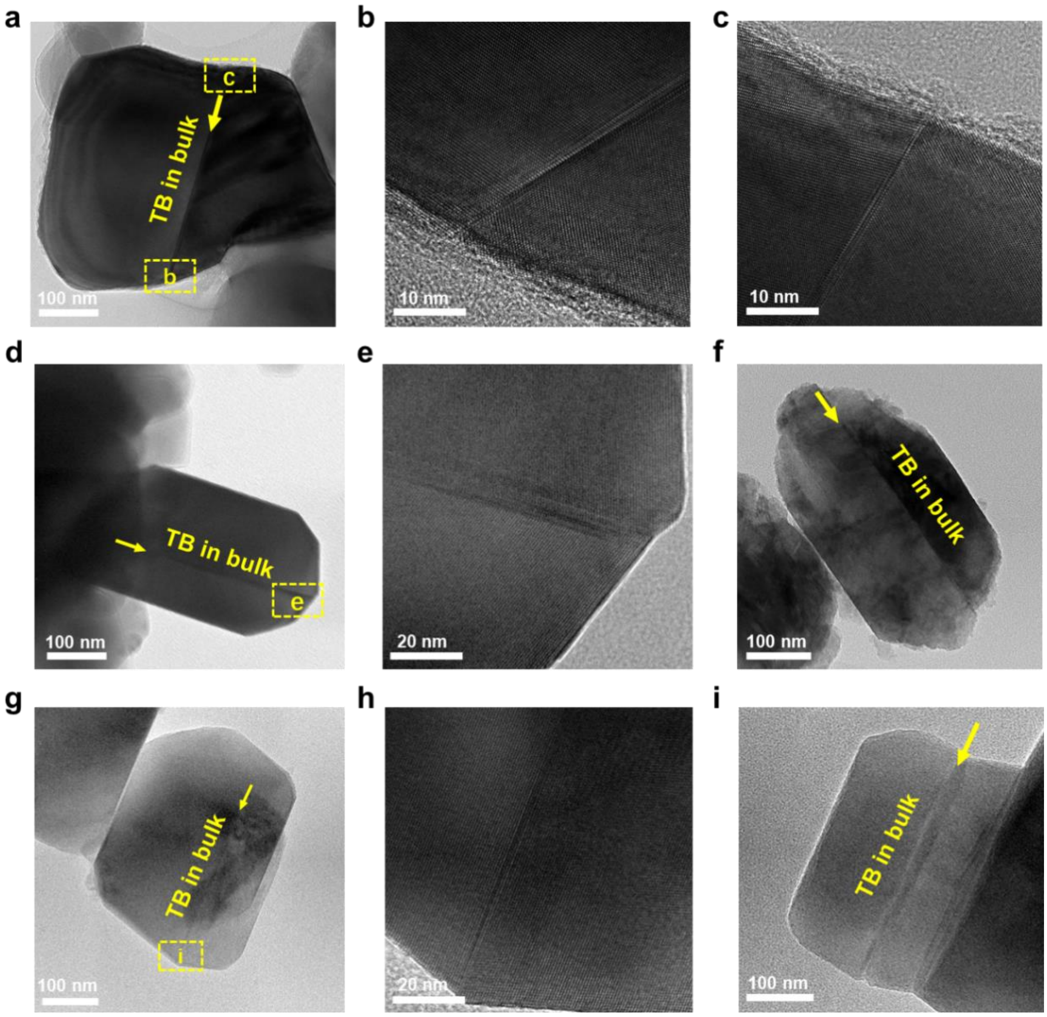

Supplementary Fig. 6: Bright-field TEM images of the twin boundary defects in the bulk LMO-TB particles.

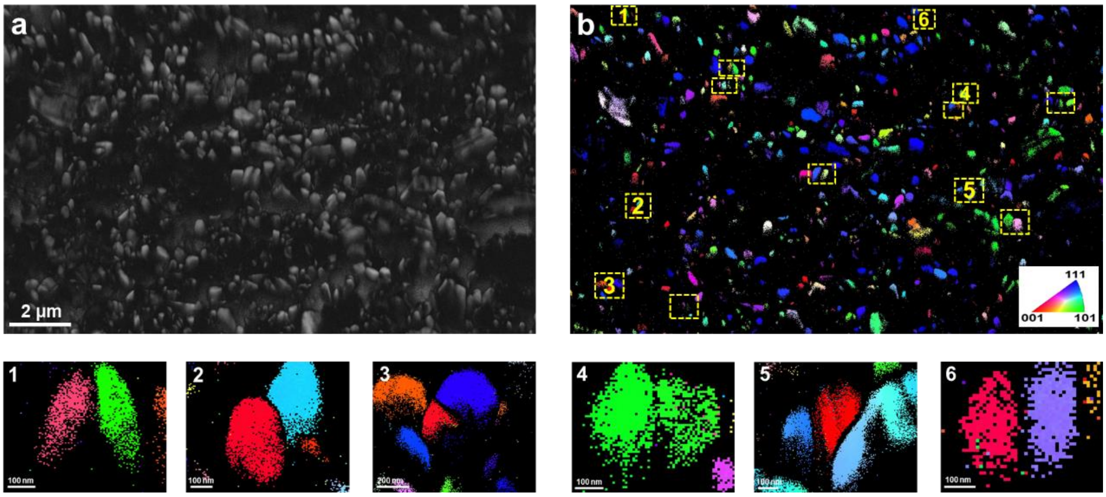

Supplementary Fig. 7: (a) Band contrast images and (b) IPF-Z images of the LMO-TB particles with twin boundaries.

In the mapping region of 17×10 μm^2, twinning particles can be easily observed, marked, magnified and exhibited. It is known that three Euler angles Ψ, θ, φ (around the z', x' and z' axes, respectively) can be used to describe the orientation of each grain with respect to the sample coordinates. By adopting matrix multiplication, the rotation can be described as:

$$\boldsymbol{E}(\psi,\theta,\varphi)=\begin{pmatrix}\cos\varphi & \sin\varphi & 0\\ -\sin\varphi & \cos\varphi & 0\\ 0 & 0 & 1\end{pmatrix}\begin{pmatrix}1 & 0 & 0\\ 0 & \cos\theta & \sin\theta\\ 0 & -\sin\theta & \cos\theta\end{pmatrix}\begin{pmatrix}\cos\psi & \sin\psi & 0\\ -\sin\psi & \cos\psi & 0\\ 0 & 0 & 1\end{pmatrix}$$

According to the HAADF STEM images in **Fig. 3** of the manuscript, the twin elements in LMO-TB are $\{111\}\langle 11\bar{2}\rangle$, which possess the orientation relationship of $[110]/70.5°$, $[111]/60°$ and $[210]/131.8°$. If we denote $\boldsymbol{R_v}(\alpha)$ as the rotation around the given crystal direction $\boldsymbol{v}$ with angle α, the orientations of the twinning particles ($\boldsymbol{E_T}$ and $\boldsymbol{E_0}$) should satisfy:

$$\boldsymbol{E_T}(\psi_T,\ \theta_T,\ \varphi_T)=\boldsymbol{E_0}(\psi_0,\ \theta_0,\ \varphi_0)\boldsymbol{R_{\langle 111\rangle}}(60°),$$
$$\boldsymbol{E_T}(\psi_T,\ \theta_T,\ \varphi_T)=\boldsymbol{E_0}(\psi_0,\ \theta_0,\ \varphi_0)\boldsymbol{R_{\langle 110\rangle}}(\mathbf{70.5°}),$$
$$\boldsymbol{E_T}(\psi_T,\ \theta_T,\ \varphi_T)=\boldsymbol{E_0}(\psi_0,\ \theta_0,\ \varphi_0)\boldsymbol{R_{\langle 210\rangle}}(131.8°).$$

Here, two typical twinning particles are enlarged and demonstrated in **Supplementary Fig. 8**. In **Supplementary Fig. 8a-c**, the $[110]$ direction of the particle is nearly parallel to the normal direction (Z-axis) of the sample plane. Therefore, the $\{110\}$ pole figure in **Supplementary Fig. 8b** displays two sets of trace points that have mirror symmetry and an angle difference of ~70°, which corresponds well with the discussion above. From the $\{111\}$ pole figure in **Supplementary Fig. 8c**, one shared trace point is readily observed, representing the twin boundary of the grains. No other shared $\{111\}$ point exists, indicating that there is only one variant in this particle. Similar results are obtained in another twinning structure, as shown in **Supplementary Fig. 8d-e**, except that the $[110]$ direction is not perpendicular to the observation plane.

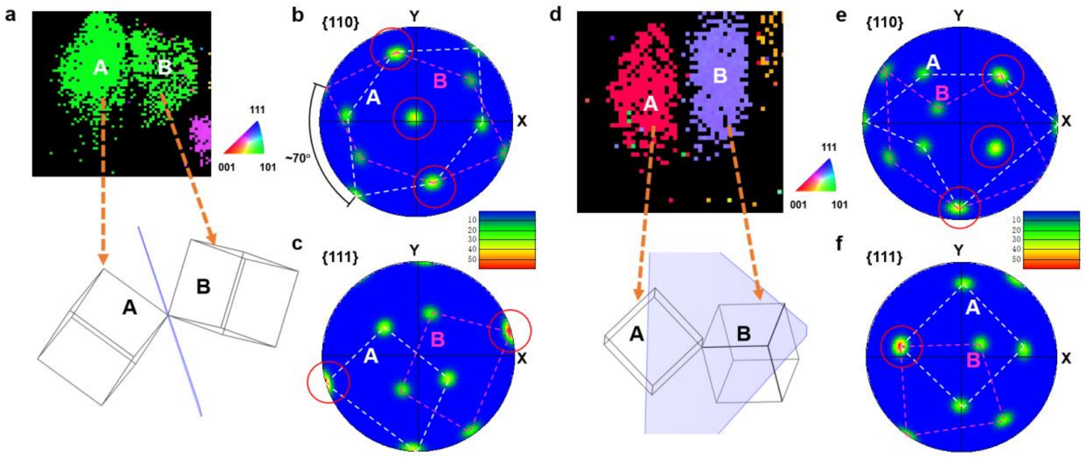

Supplementary Fig. 8: (a) IPF-Z image of one particle of LMO-TB. Cubes marked A and B represent the orientation obtained from the Tango Software of regions A and B, which were split by a twinning boundary in a single particle. (b) Pole figures in {110} of the two regions. (c) Pole figures in {111} of the two regions. (d)-(f) Another particle containing twinning in the LMO-TB sample.

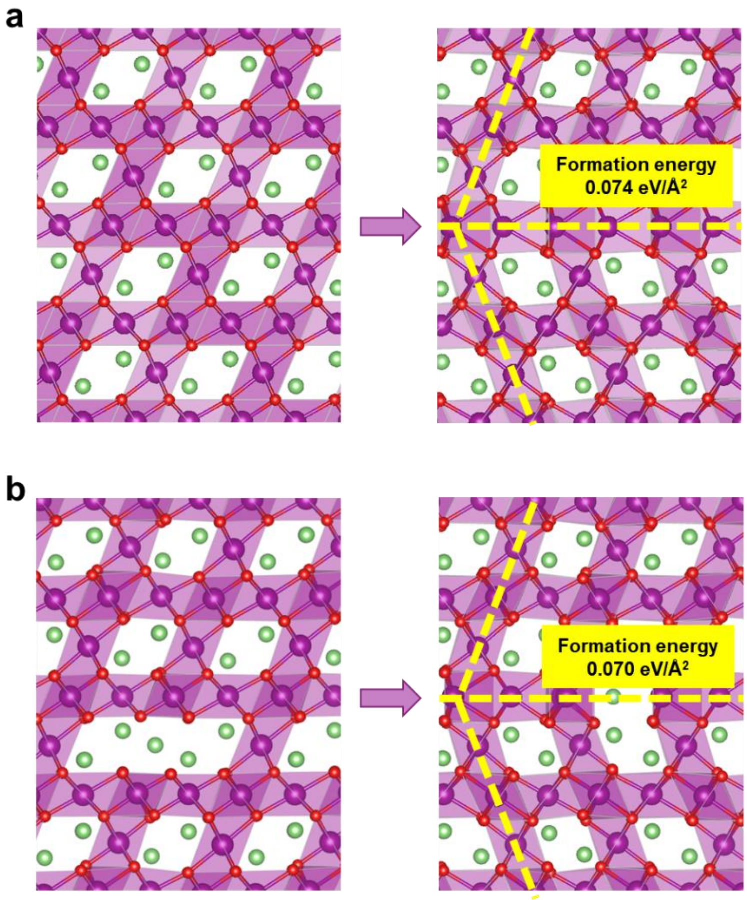

Supplementary Fig. 9: (a) Illustration of the bulk and symmetrical twin boundary of LMO. (c) Illustration of the bulk and symmetrical twin boundary of LMO-TB.

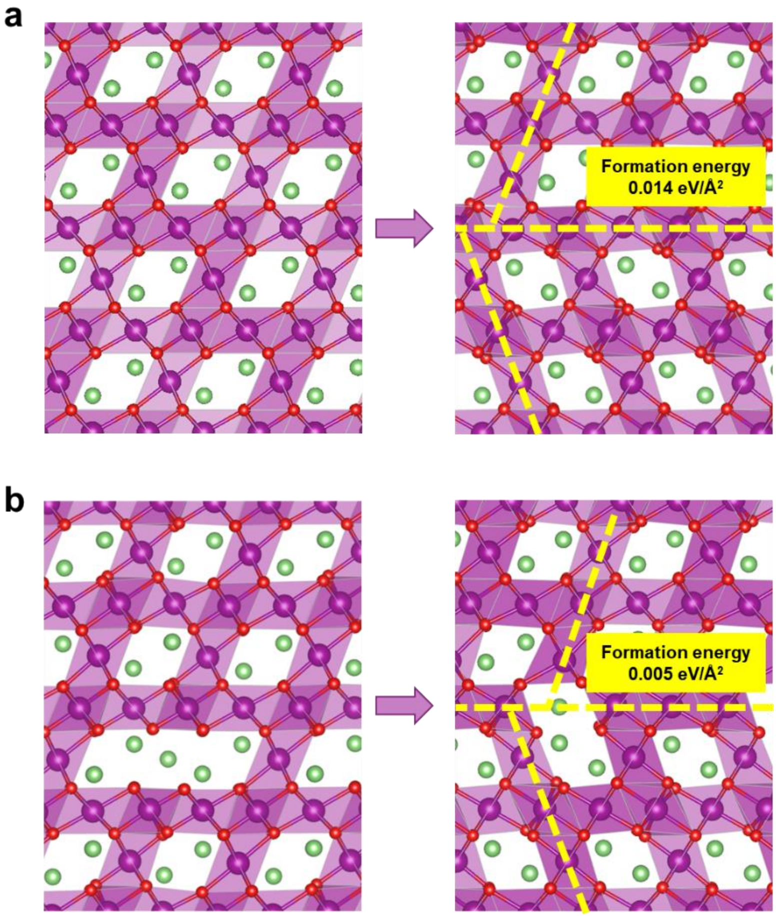

Supplementary Fig. 10: (a) Illustration of the bulk and asymmetrical twin boundary of LMO. (c) Illustration of the bulk and asymmetrical twin boundary of LMO-TB.

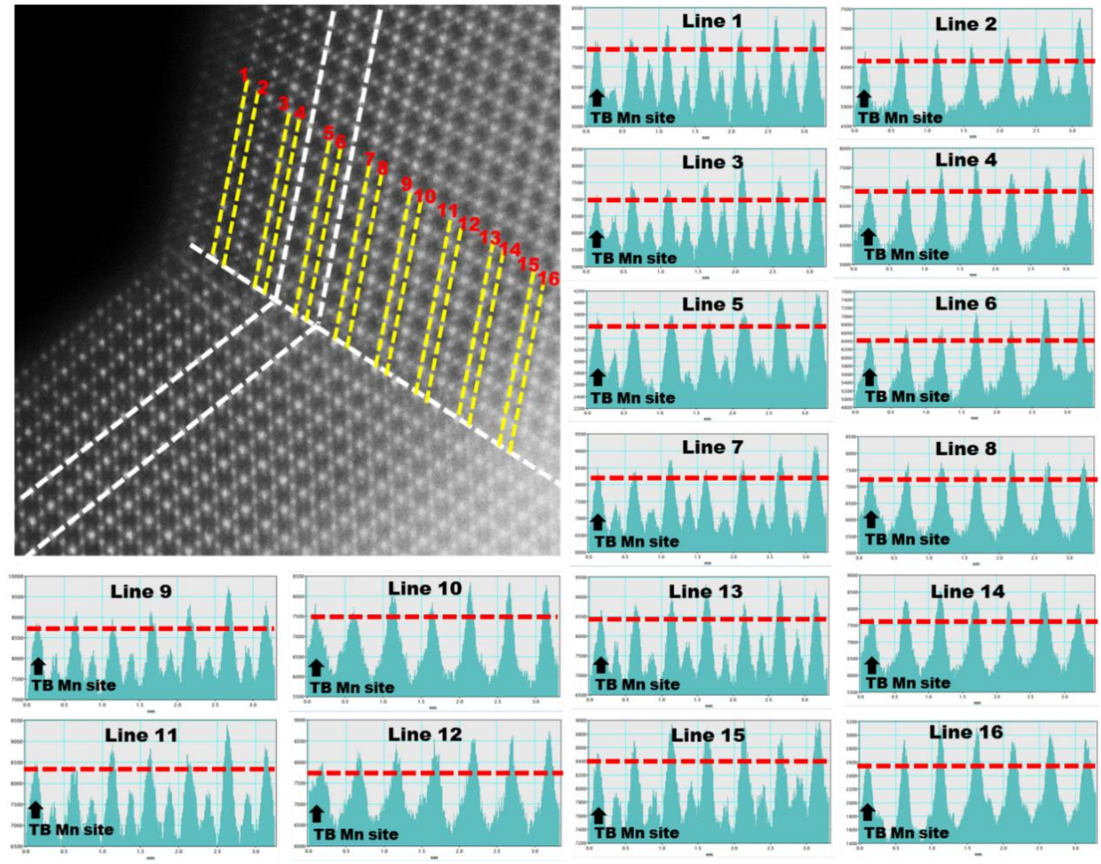

Supplementary Fig. 11: Atomically resolved HAADF STEM image of the symmetrical boundaries and corresponding atomic contrast curves of line 1 to line 16. In each atomic contrast curve, the first peak, which was marked with the black arrow, represented the Mn atom contrast in the symmetrical boundaries.

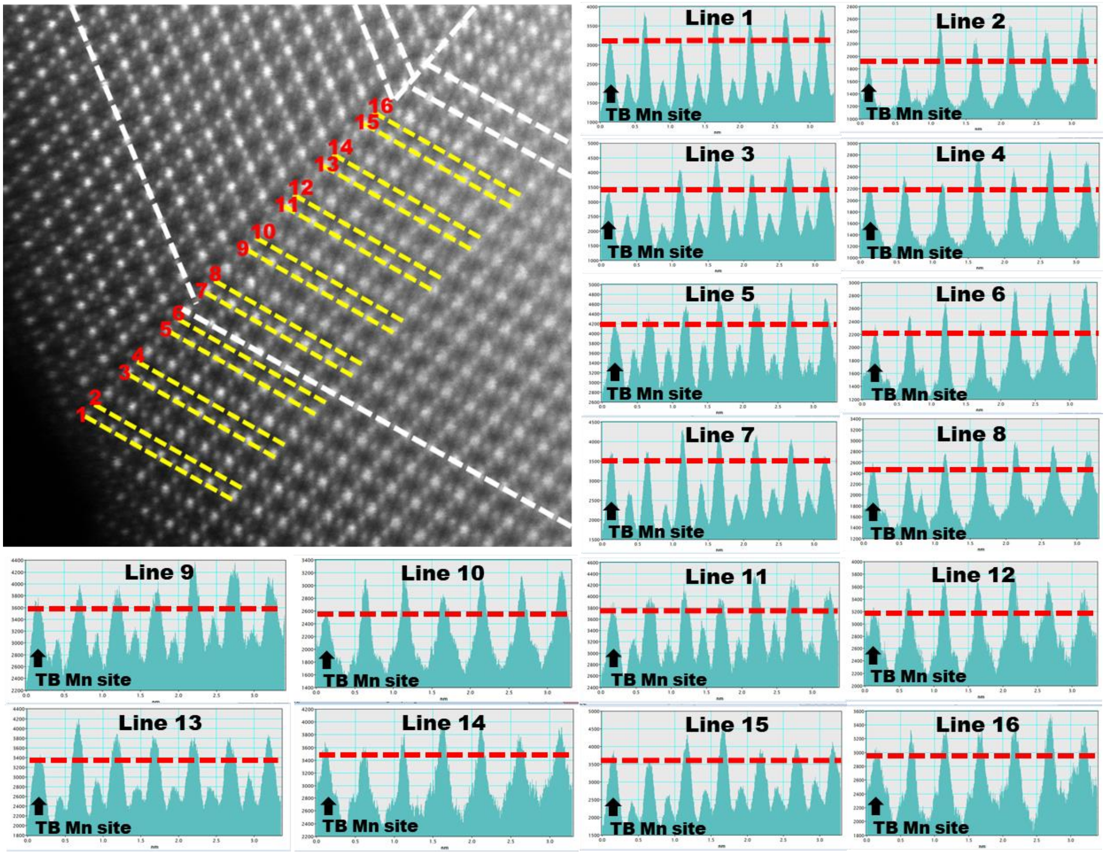

Supplementary Fig. 12: Atomically resolved HAADF STEM image of the asymmetrical boundaries and corresponding atomic contrast curves of line 1 to line 16. In each atomic contrast curve, the first peak, which was marked with the black arrow, represented the Mn atom contrast in the asymmetrical boundaries.

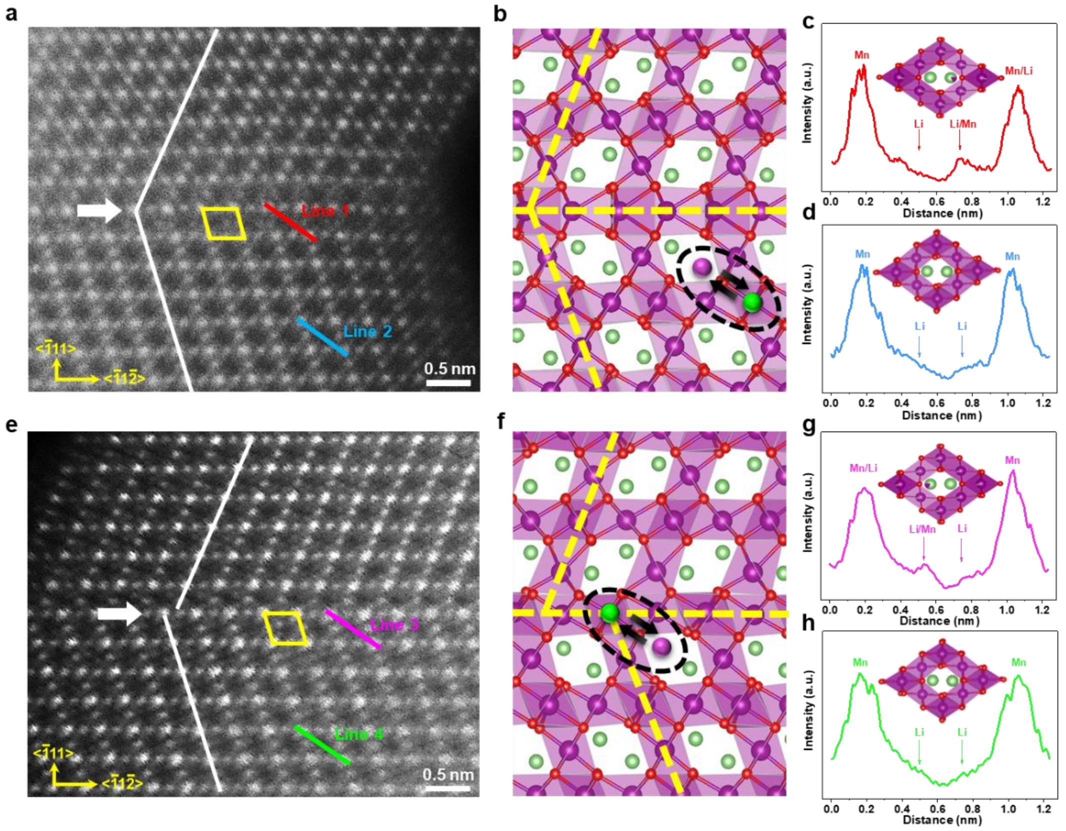

Supplementary Fig. 13: (a) Atomically resolved HAADF STEM image and a (b) schematic of the symmetrical twin boundaries. (c, d) Corresponding atomic contrast curves of Line 1 and Line 2 in (a). (e) Atomically resolved HAADF STEM image and a (f) schematic of the asymmetrical boundaries. (g, h) Corresponding atomic contrast curves of Line 3 and Line 4 in (e).

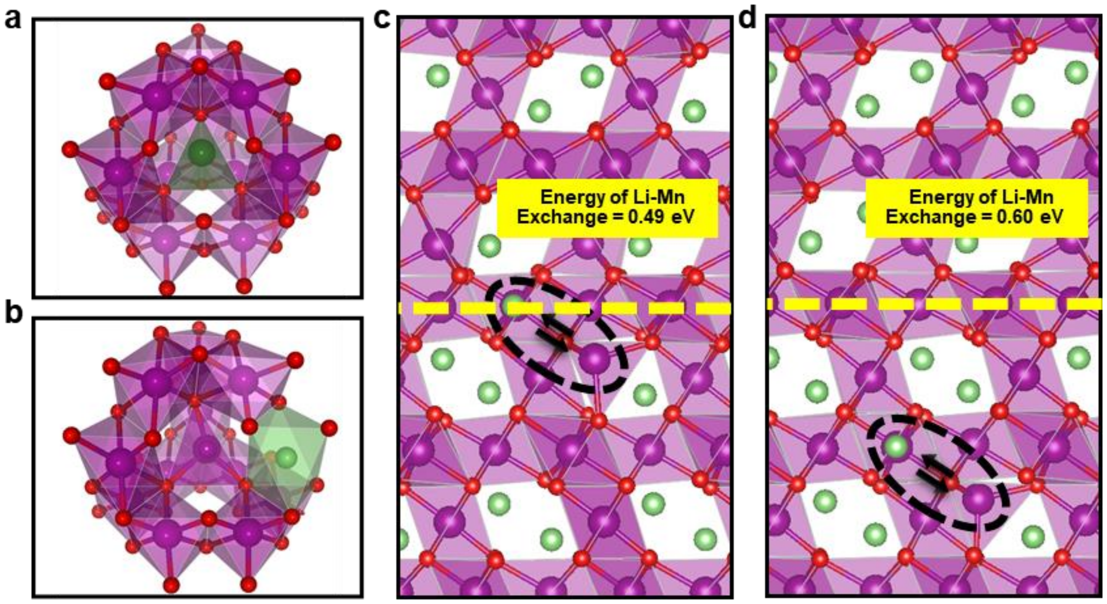

Supplementary Fig. 14: (a) Illustration of $LiMn_2O_4$ (the coordination geometry around the Li atom is a tetragonal pyramid with 12 equivalent Mn atoms). (b) Illustration of the Li-Mn exchange in $LiMn_2O_4$. (c) Li-Mn exchange of LMO-TB around the asymmetrical twinning structure. (d) Li-Mn exchange of LMO-TB in the bulk.

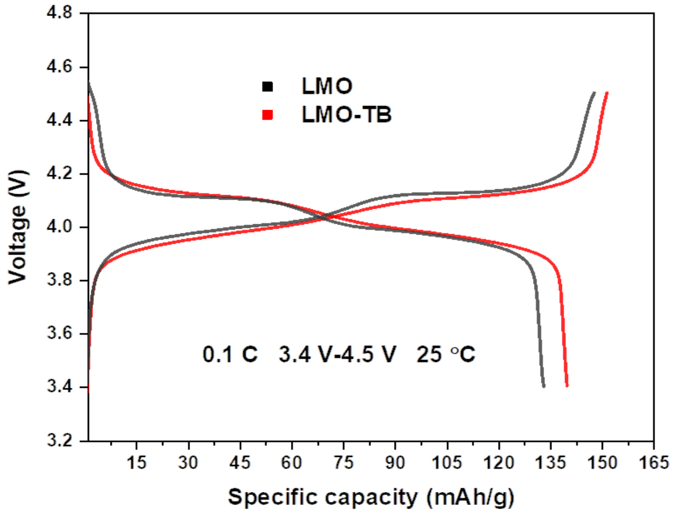

Supplementary Fig. 15: Charge-discharge curves at a rate of 0.1C in a voltage window of 3.4-4.5 V for LMO and LMO-TB.

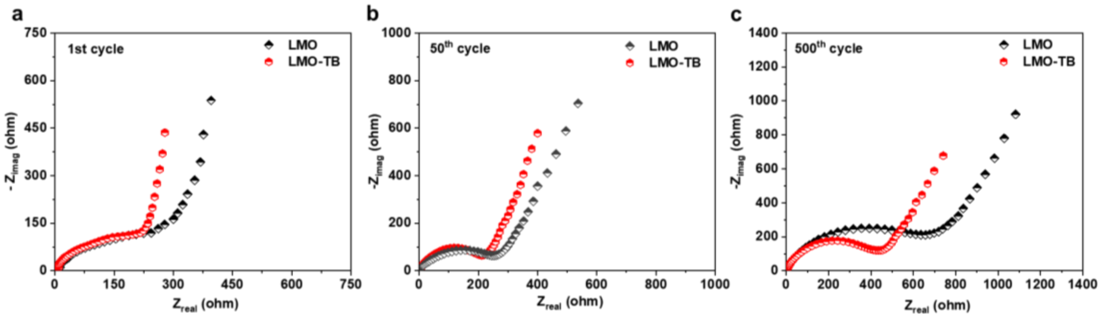

Supplementary Fig. 16: Nyquist plots after (a) the 1st cycle, (d) 50th cycle, and (e) 500th cycle of LMO and LMO-TB.

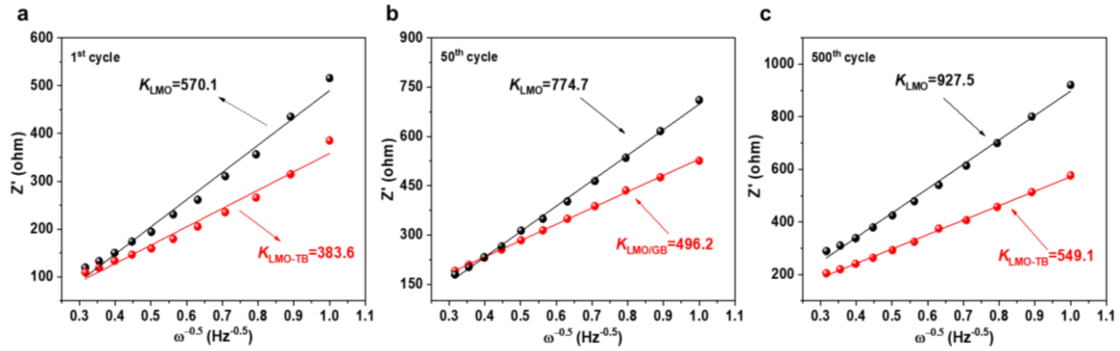

Supplementary Fig. 17: Z'-$\omega^{-1/2}$ diagram calculated from the impedance spectroscopy of LMO and LMO-TB.

At low frequencies, the Li^+ diffusion coefficient formula can be expressed as follows[1,2]:

$$D_{Li+} = R^2T^2/2A^2n^4F^4C^2A_w^2 \qquad (1)$$

where R is the gas constant, T is the Kelvin temperature, A is the electrode surface area, n is the number of transferred electrons per mole in the electrode reaction, C is the concentration of lithium in the electrode, and A_w is the Warburg coefficient. In addition, the Warburg coefficient A_w has the following relationship[1,2]:

$$Z' = R_S + R_{ct} + A_w\omega^{-1/2} \qquad (2)$$

where R_S is the solution resistance and R_{ct} is the charge-transfer reaction resistance. Therefore, the value of the Warburg coefficient can be obtained from the Z'-$\omega^{-1/2}$ diagram, and the slope fitted from the Z'-$\omega^{-1/2}$ diagram is the Warburg coefficient A_w. According to equation (1) and the Warburg coefficient A_w, we can obtain the lithium-ion diffusion rate D_{Li+}.

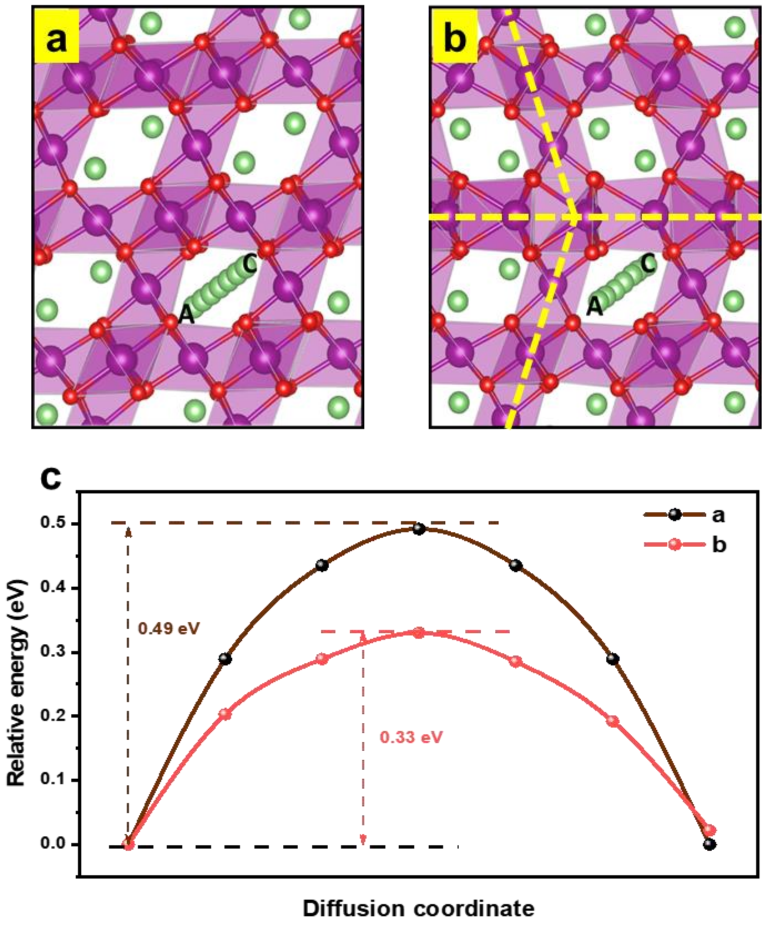

Supplementary Fig. 18: Schematic diagram of lithium-ion diffusion in the (a) bulk structure and (b) symmetrical twin boundary. (c) Energy barrier of lithium-ion diffusion under the conditions of (a) and (b).

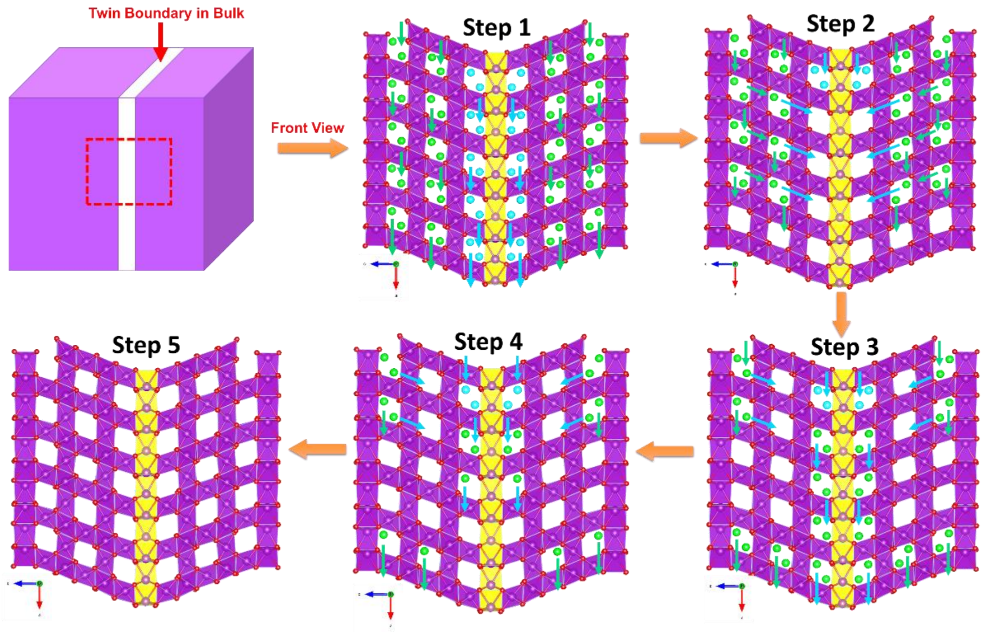

Supplementary Fig. 19: Schematic diagram of the lithium-ion diffusion process in LMO-TB.

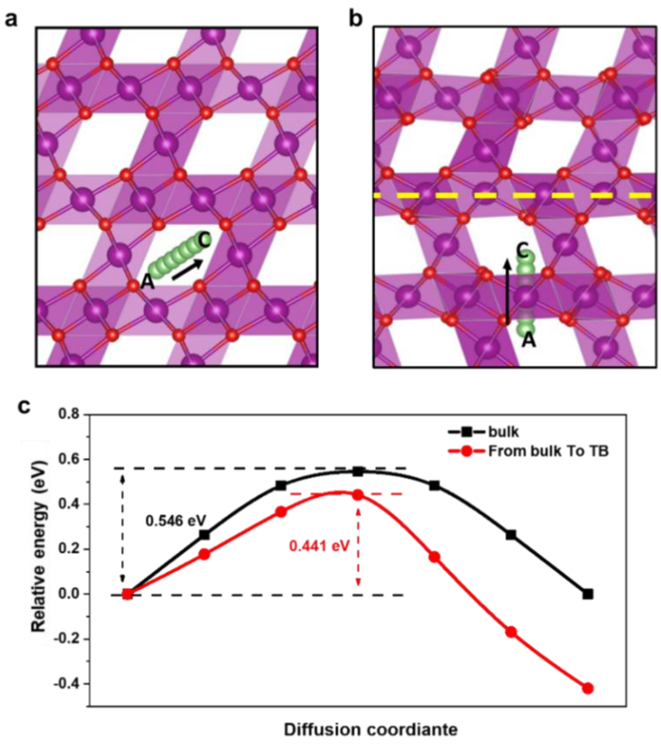

Supplementary Fig. 20: Schematic diagram of the Li-ion diffusion process in the (a) bulk and (b) from the bulk to the twin boundary. (c) Energy barrier of lithium-ion diffusion under the two conditions of (a) and (b).

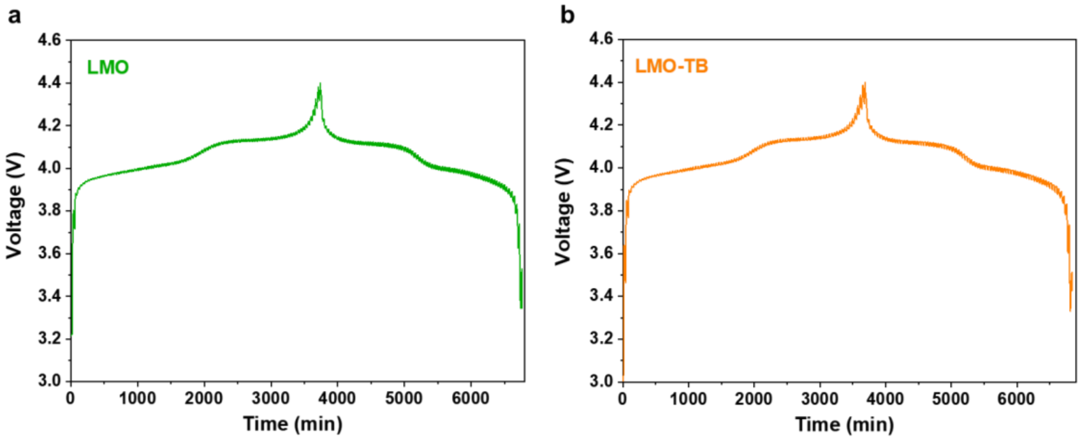

Supplementary Fig. 21: GITT curves of (a) LMO and (b) LMO-TB during the first cycle.

The Li^+ diffusion rate calculated from the GITT curve is based on the following formula according to Fick's second law of diffusion[3,4]:

$$D_S = \frac{4}{\pi\tau}\left(\frac{n_M V_M}{S}\right)^2 \left(\frac{\Delta E_S}{\Delta E_t}\right)^2 \quad (3)$$

where τ is the duration of the current pulse, n_M is the number of moles, V_M is the molar volume of the electrode, S is the electrode-electrolyte contact area, and ΔE_s and ΔE_t are the change in the steady state potential and the total change during the current flux by deducting the IR drop, respectively.

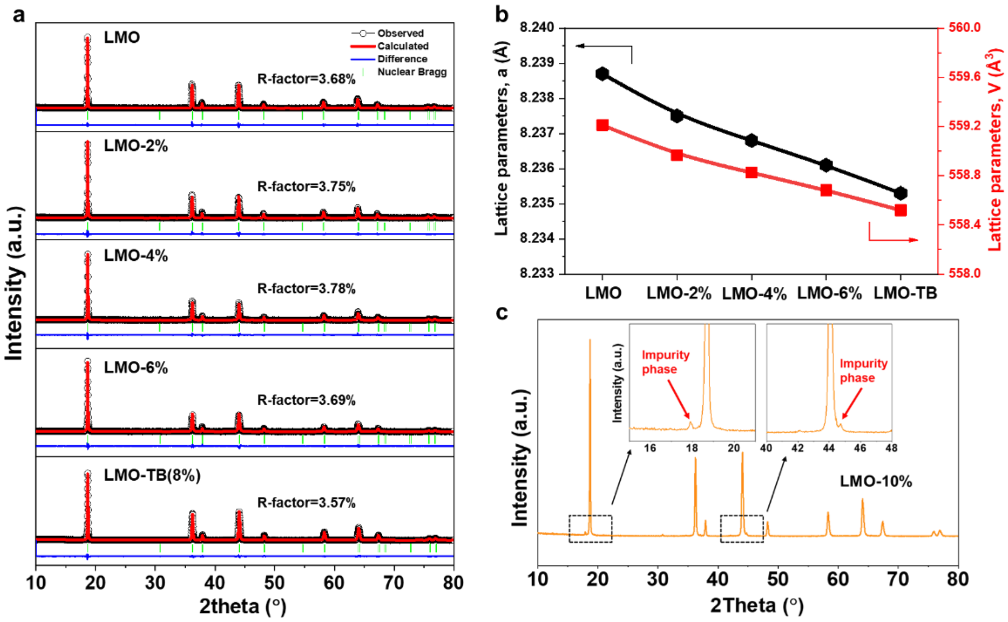

Supplementary Fig. 22: (a) Powder XRD refinement patterns for LMO, LMO-2%, LMO-4%, LMO-6% and LMO-8% (LMO-TB). (b) Lattice parameters a and V of LMO, LMO-2%, LMO-4%, LMO-6% and LMO-8% (LMO-TB). (c) Powder XRD diffraction pattern of LMO-10%.

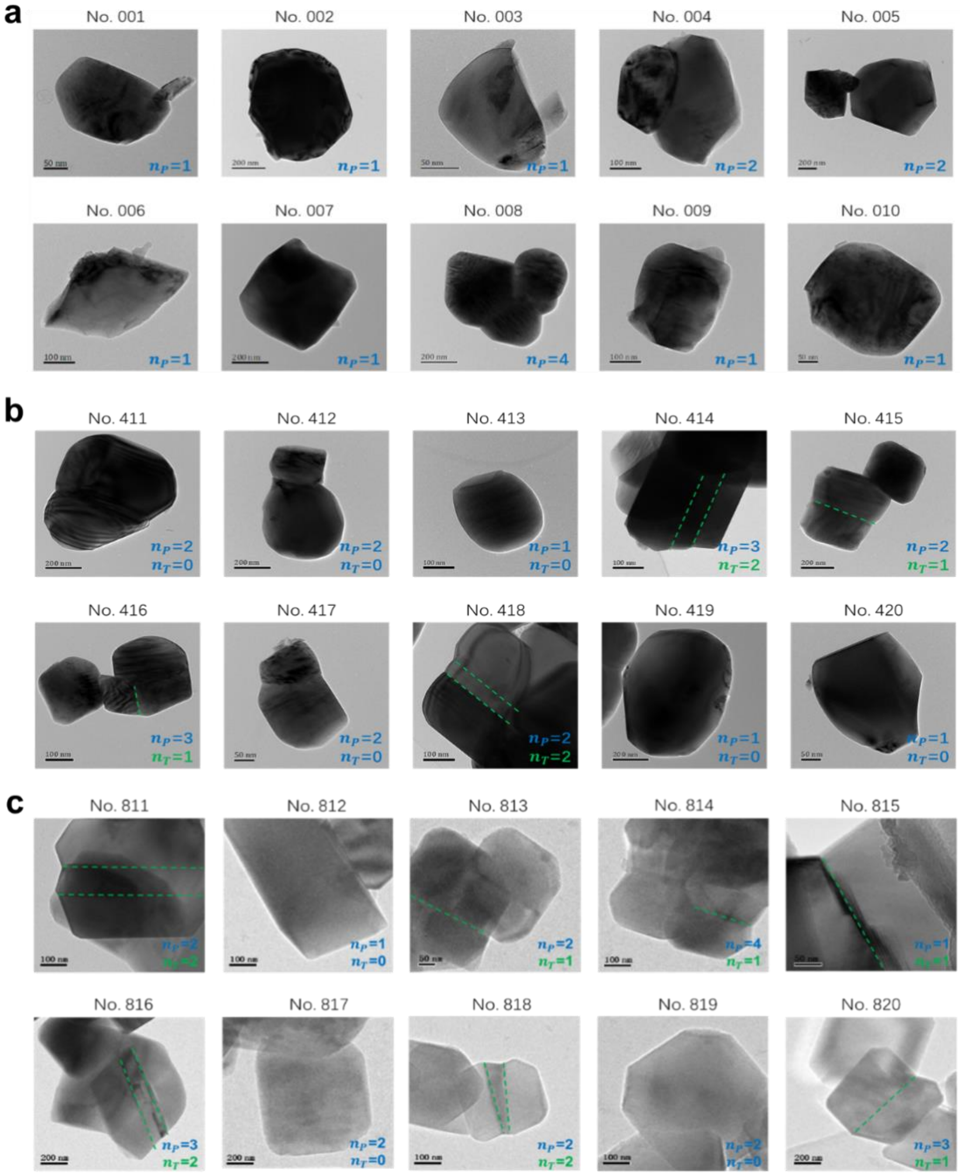

Supplementary Fig. 23: (a) Representative TEM images of the LMO particles (No. 001-No. 010). (b) Representative TEM images of the LMO-4% particles (No. 411-No. 420). (c) Representative TEM images of the LMO-TB particles (No. 811-No. 820).

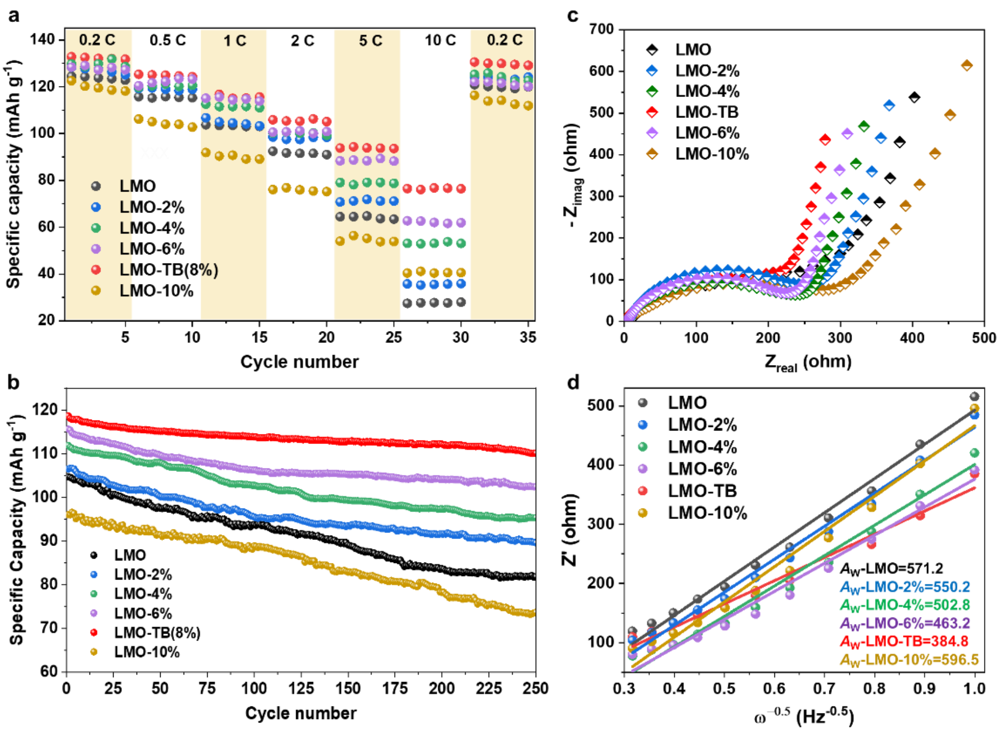

Supplementary Fig. 24: (a) Rate capacities and (b) long-term cycling of LMO, LMO-2%, LMO-4%, LMO-6%, LMO-8% (LMO-TB) and LMO-10%. (c) Nyquist plots after the first cycle and (b) Z'-$\omega^{-1/2}$ diagram calculated from the impedance spectra of LMO, LMO-2%, LMO-4%, LMO-6%, LMO-8% (LMO-TB) and LMO-10%. A_w is the Warburg coefficient.

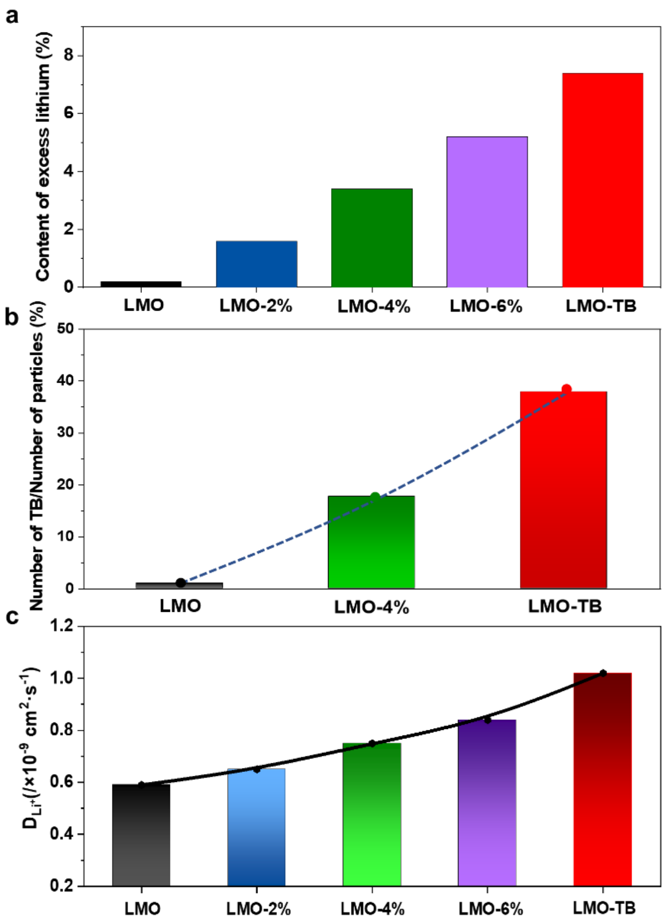

Supplementary Fig. 25: (a) Content of excess lithium of LMO, LMO-2%, LMO-4%, LMO-6% and LMO-8% (LMO-TB), as calculated by ICP-AES. (b) Calculated values of the number of twin boundaries divided by the number of particles measured in LMO, LMO-4% and LMO-8% (LMO-TB) according to the statistics from the TEM images. (c) Diffusion coefficients of the lithium ions, as calculated from the impedance spectra.

Supplementary Discussion

Systematic Analysis of the compositions, defects and properties. To further validate the positive correlations between the amount of excess Li, contents of defects and lithium-ion diffusion kinetics in spinel LMO material, a batch of $Li_{1+\delta}Mn_2O_4$ samples with different contents of excess Li, i.e., δ = 0% (LMO), 2%, 4%, 6%, 8% (LMO-TB), and 10%, were studied. The XRD (**Supplementary Fig. 22**) patterns show that the samples with a Li excess below 8% are all single phases, whereas a small number of impurities appears in that with a Li excess equal to 10%, indicating that the limit of excess lithium is 8%. Based on the Rietveld refinement results, the lattice constants of all samples are deduced, and the lattice constant of samples with the pure spinel phase decreases in a monotonic manner upon the integration of excess lithium. This result signals not only a shrinkage in the unit cell but also the successful integration of different amounts of excessive Li^+ into the structural lattice.

Consequently, additional bright-field TEM experiments were performed on samples with three representative contents of excess Li, i.e., Li-excess δ = 0%, 4%, and 8%, respectively. All the bright-field STEM images of the stoichiometric LMO sample (**Supplementary Fig. 23a**) show that twin boundaries can hardly be observed, although more than 60 particles are studied and these particles exhibit various morphologies. In contrast, 11 twin boundaries are found in 62 measured particles measured in the LMO-4% sample (**Supplementary Fig. 23b**), yielding a calculated ratio (numbers of twin boundaries divided by the numbers of particles measured) of approximately 17.7%. As for the LMO-8% sample, i.e., LMO-TB, this ratio further increases to approximately 37.9% (**Supplementary Fig. 23c**). Clearly, the concentration of twin boundaries in the LMO-8% sample is higher than that in the LMO-4% sample, and quite a few particles possess more than one twin boundary. It should be noted that for randomly dispersed particles, especially with a large amount of particle agglomerations and with tilted twinning boundaries that may not be seen under a fixed incident electron beam, quantitative measurements of the twin boundary ratio are challenging, if not even impossible. Nevertheless, according to the statistics listed above, it can be fairly concluded that the number of planar defects, i.e., twin boundaries, is consistent with the amount of excess Li.

Next, the electrochemical properties of the $Li_{1+\delta}Mn_2O_4$ samples were evaluated. First, **Supplementary Fig. 24a** shows that the specific capacities at all rates (0.2, 0.5, 1, 2, 5 and 10C) increase with the content of TB defects in the range of a Li excess δ = 0% to 8%. Once the amount of excess lithium is further increased up to 10%, the rate

capacities decrease due to poor lithium-ion diffusion in the newly formed $Li_2Mn_2O_4$ impurity [5]. In addition, **Supplementary Fig. 24b** shows that the appearance of an impurity phase in the LMO-10% sample decreases the cycling life of the battery. Furthermore, EIS of all $Li_{1+\delta}Mn_2O_4$ samples under the same battery conditions was performed (**Supplementary Fig. 24c**). On the basis of the fitting results at low frequency (**Supplementary Fig. 24d**), the lithium-ion diffusion coefficients of $Li_{1+\delta}Mn_2O_4$ (δ = 0% (LMO), 2%, 4%, 6%, and 8% (LMO-TB)) samples are deduced to be 0.59×10^{-9}, 0.65×10^{-9}, 0.75×10^{-9}, 0.84×10^{-9}, and 1.02×10^{-9} cm^2 s^{-1}, respectively (**Supplementary Fig. 25**), indicating the gradual increase in diffusion kinetics with an increasing amount of excess lithium and an increasing concentration of twin boundaries. In summary, the LMO-TB sample possesses the best electrochemical performance, which can be attributed to the generation of the largest content of TB defects. The results of LMO-TB can be well explained within the framework of twin boundary defect engineering.

Supplementary References

1. Zheng, J., Yan, P., Estevez, L., Wang, C. & Zhang, J. G. Effect of calcination temperature on the electrochemical properties of nickel-rich $LiNi_{0.76}Mn_{0.14}Co_{0.10}O_2$ cathodes for lithium-ion batteries. *Nano Energy* **49**, 538-548, (2018).

2. Yang, J., Kang, X., Hu, L., Gong, X. & Mu, S. Nanocrystalline-Li_2FeSiO_4 synthesized by carbon frameworks as an advanced cathode material for Li-ion batteries. *J. Mater. Chem. A* **2**, 6870-6878, (2014).

3. Kong, D. *et al.* Ti-Gradient Doping to Stabilize Layered Surface Structure for High Performance High-Ni Oxide Cathode of Li-Ion Battery. *Adv. Energy Mater.* **9**, (2019).

4. Zhou, P. *et al.* Synthesis, structure, and electrochemical properties of O3-type monoclinic $NaNi_{0.8}Co_{0.15}Al_{0.05}O_2$ cathode materials for sodium- ion batteries. *J. Mater. Chem. A* **7**, 657-663, (2019).

5. Zhu, X. *et al.* $LiMnO_2$ cathode stabilized by interfacial orbital ordering for sustainable lithium-ion batteries. *Nat. Sustain.* (2020)
